# Genetic association between germline *JAK2* polymorphisms and myeloproliferative neoplasms in Hong Kong Chinese population: a case–control study

**DOI:** 10.1186/s12863-014-0147-y

**Published:** 2014-12-20

**Authors:** Su Pin Koh, Shea Ping Yip, Kwok Kuen Lee, Chi Chung Chan, Sze Man Lau, Chi Shan Kho, Chi Kuen Lau, Shek Ying Lin, Yat Ming Lau, Lap Gate Wong, Ka Leung Au, Kit Fai Wong, Raymond W Chu, Pui Hung Yu, Eudora YD Chow, Kate FS Leung, Wai Chiu Tsoi, Benjamin YM Yung

**Affiliations:** Department of Health Technology and Informatics, The Hong Kong Polytechnic University, Hong Kong, China; Departments of Medicine, Princess Margaret Hospital, Hong Kong, China; Department of Medicine, Queen Elizabeth Hospital, Hong Kong, China; Department of Medicine, Pamela Youde Nethersole Eastern Hospital, Hong Kong, China; Department of Medicine, Tseung Kwan O Hospital, Hong Kong, China; Department of Medicine & Geriatrics, United Christian Hospital, Hong Kong, China; Departments of Pathology, Princess Margaret Hospital, Hong Kong, China; Departments of Pathology, Queen Elizabeth Hospital, Hong Kong, China; Departments of Pathology, Pamela Youde Nethersole Eastern Hospital, Hong Kong, China; Departments of Pathology, Tseung Kwan O Hospital, Hong Kong, China; Departments of Pathology, United Christian Hospital, Hong Kong, China; Departments of Pathology, North District Hospital, Hong Kong, China; Hong Kong Red Cross Blood Transfusion Service, Hong Kong, China

**Keywords:** Myeloproliferative neoplasms, Janus Kinase 2 (*JAK2*), *V617F* mutation, Single nucleotide polymorphisms, Genetic susceptibility

## Abstract

**Background:**

Myeloproliferative neoplasms (MPNs) are a group of haematological malignancies that can be characterised by a somatic mutation (*JAK2V617F*). This mutation causes the bone marrow to produce excessive blood cells and is found in polycythaemia vera (~95%), essential thrombocythaemia and primary myelofibrosis (both ~50%). It is considered as a major genetic factor contributing to the development of these MPNs. No genetic association study of MPN in the Hong Kong population has so far been reported. Here, we investigated the relationship between germline *JAK2* polymorphisms and MPNs in Hong Kong Chinese to find causal variants that contribute to MPN development. We analysed 19 tag single nucleotide polymorphisms (SNPs) within the *JAK2* locus in 172 MPN patients and 470 healthy controls. Three of these 19 SNPs defined the reported *JAK2* 46/1 haplotype: rs10974944, rs12343867 and rs12340895. Allele and haplotype frequencies were compared between patients and controls by logistic regression adjusted for sex and age. Permutation test was used to correct for multiple comparisons. With significant findings from the 19 SNPs, we then examined 76 additional SNPs across the 148.7-kb region of *JAK2* via imputation with the SNP data from the 1000 Genomes Project.

**Results:**

In single-marker analysis, 15 SNPs showed association with *JAK2V617F*-positive MPNs (n = 128), and 8 of these were novel MPN-associated SNPs not previously reported. Exhaustive variable-sized sliding-window haplotype analysis identified 184 haplotypes showing significant differences (*P* < 0.05) in frequencies between patients and controls even after multiple-testing correction. However, single-marker alleles exhibited the strongest association with *V617F*-positive MPNs. In local Hong Kong Chinese, rs12342421 showed the strongest association signal: asymptotic *P* = 3.76 × 10^−15^, empirical *P* = 2.00 × 10^−5^ for 50,000 permutations, OR = 3.55 for the minor allele *C*, and 95% CI, 2.59-4.87. Conditional logistic regression also signified an independent effect of rs12342421 in significant haplotype windows, and this independent effect remained unchanged even with the imputation of additional 76 SNPs. No significant association was found between *V617F*-negative MPNs and *JAK2* SNPs.

**Conclusion:**

With a large sample size, we reported the association between *JAK2V617F*-positive MPNs and 15 tag *JAK2* SNPs and the association of rs12342421 being independent of the *JAK2* 46/1 haplotype in Hong Kong Chinese population.

**Electronic supplementary material:**

The online version of this article (doi:10.1186/s12863-014-0147-y) contains supplementary material, which is available to authorized users.

## Background

Myeloproliferative neoplasms (MPNs) are a group of clonal diseases originating from the bone marrow. The present study focuses on three main MPNs: polycythaemia vera (PV), essential thrombocythaemia (ET), and primary myelofibrosis (PMF) [1]. These three non-leukaemic MPNs are characterised by their BCR-ABL-negativity and recurrent genetic aberrations, particularly a somatic mutation, *JAK2V617F* (hereafter *V617F*). This point mutation leads to the Val-to-Phe substitution at the amino acid position 617 and constitutively activates the JAK-STAT signalling pathway that is essential for homeostatic processes including proliferation and survival of haematopoietic cells [2,3]. It was detected in almost all PV patients and about half of ET and PMF patients, but not in healthy individuals [4-7]. In 2008, World Health Organization included *V617F* as one of the diagnostic criteria for this group of MPNs [1]. Subsequently, disease anticipation was first reported in Swedish families with an increased risk of developing MPNs among the first-degree relatives of MPN patients [8]. Thereafter, more MPN predisposition loci were revealed by several independent groups around the same time. It was found that the *JAK2* germline haplotype 46/1 increased the likelihood of developing MPNs, mainly in patients with the *JAK2* mutation [9-15]. Association of *JAK2* alleles and/or haplotypes with MPNs has now been reported in Caucasians [9-13,16-18], Japanese [14,15], Chinese [19-22] and Brazilians [23]. However, work remains to be done to identify the causal variants in or flanking the *JAK2* locus and to delineate the mechanism by which such casual variants contribute to MPN development.

The aim of this study was to evaluate the association between *JAK2* germline polymorphisms and MPNs in the Chinese population of Hong Kong. Our primary hypothesis was that the disease might have possible association with *other* variants spanning the *JAK2* gene. Our case–control association study was carried out in two stages on the same sample set (n = 642): an initial direct genotyping of 19 SNPs including the reported *JAK2* 46/1 risk-haplotype-tagging SNPs and other tag SNPs selected from HapMap [24], and an imputation study of additional 76 SNPs in an attempt to narrow down the targeted region involved in the development of MPNs. Among Asian studies, we have the largest sample size of controls (n = 470) and the second largest total sample size (n = 642).

## Results

### Participants

We recruited 172 MPN patients and 470 healthy control subjects, all Chinese. The patients included 61 with PV, 93 with ET, 17 with PMF, and 1 with unclassified MPN, and 86 males (50.0%) and 86 females (50.0%). Their mean age was 57 years (ranges: 18–88 years). For the healthy controls, the mean age was 51 years (ranges: 16–75 years), and there were 236 males (50.2%) and 234 females (49.8%).

### Detection of *JAK2V617F* mutation in Hong Kong Chinese

All cases and controls were first screened for *V617F* mutation. Overall, 128 (74.4%) MPN patients were positive and 44 (25.6%) negative for *V617F*. Age differed significantly between *V617F*-positive MPN cases and healthy controls (*P* < 0.0001) whereas there was no difference in age between *V617F*-negative MPNs and controls (*P* = 0.7342). However, there was still statistically significant difference in age between all MPN cases (both *V617F*-positive and -negative) and controls (*P* < 0.0001). Fisher’s exact test suggested no significant difference in sex ratio between the two groups (*P* > 0.3). The prevalence of *V617F* in our cohort was 87% (53/61) in PV, 68% (63/93) in ET, 65% (11/17) in PMF, and 100% (1/1) in unclassified MPN. The mutation frequency did not differ by sex and age in our patient group. Overall, the data suggested that MPNs can affect anyone regardless of sex and age, in our Hong Kong Chinese population. The mutation was not detected in the 470 healthy controls.

### Genetic association study of genotyped SNPs

In total, 19 tag SNPs were selected, capturing the genetic information of 95 SNPs in the study region (148.7 kb) with a mean r^2^ of 0.96. All of them are intronic SNPs except rs3808850 (5’ upstream). As explained in the section of Materials and methods, *JAK2* risk-haplotype-tagging SNPs were forced to be included. The SNPs were also called S1, S2, …., and S19 in the sequential order from the 5’ end to the 3’ end of the *JAK2* sense strand for ease of discussion.

**Figure 1:**
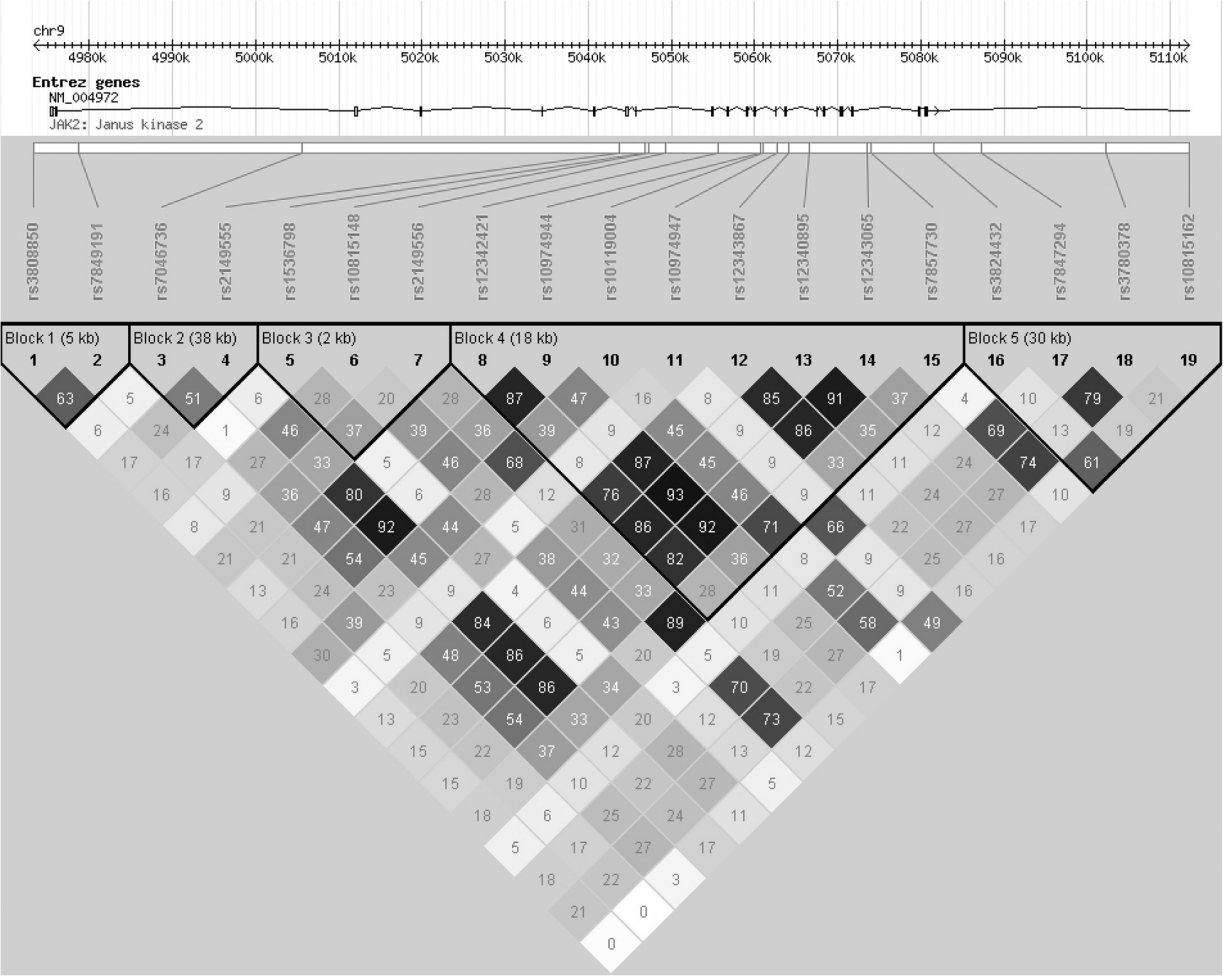
**Linkage disequilibrium pattern for 19** ***JAK2*** **SNPs for** ***V617F*** **-positive MPN cases and healthy controls.** Linkage disequilibrium plots were generated utilising the Haploview software. The values in the boxes indicate the r^2^ values between the respective pairs of SNPs and the empty boxes represent those with r^2^ = 1.0. Haplotype blocks were defined by solid spine of linkage disequilibrium.

The genotypes were in Hardy-Weinberg equilibrium (Fisher’s exact test *P* > 0.05) for all SNPs in the control group. In general, linkage disequilibrium (LD) among the 19 SNPs in the combined group of *V617F*-positive MPN cases and healthy controls was not strong except for those tagging the *JAK2* risk-haplotype (Figure 1). The same applied to the LD measures (r^2^) for the combined group of *V617F*-negative MPN cases and healthy controls (details not shown).

As a difference in age was observed between cases and controls, we sought to minimise the influence of age. To be consistent with previous studies [13,20], we also adjusted for sex in the analyses although the difference in sex ratio between cases and controls did not reach statistical significance. Among the five genetic models tested (genotypic, additive, allelic, dominant and recessive) for the 19 directly genotyped SNPs, the allelic model generated the most significant results. Therefore, we increased the stringency of our allelic test by comparing the 19 SNPs between *V617F*-positive MPNs and controls with adjustment for sex and age, and with correction for multiple comparisons by 50,000 permutations. All 19 SNPs were associated with *V617F*-positive MPNs before permutation except rs1536798 (S5; *P*_*asym*_ = 0.0765) and rs10974947 (S11; *P*_*asym*_ = 0.1414) while 2 other SNPs (rs10815148 (S6) and rs3824432 (S16)) did not survive after 50,000 permutations with *P*_*emp*_ > 0.05 (Table 1); asymptotic *P* value is denoted as *P*_*asym*_ and empirical *P* value as *P*_*emp*_. Moreover, 8 of these 15 MPN-associated SNPs were novel and have not been reported previously: rs2149555 (S4), rs2149556 (S7), rs10119004 (S10), rs12343065 (S14), rs7857730 (S15), rs7847294 (S17), rs3780378 (S18) and rs10815162 (S19) (see footnote a of Table 1).

**Table 1:** **Allelic association tests for 19 genotyped tag SNPs of the** ***JAK2*** **gene in** ***V617F*** **-positive MPNs**

	**Alleles** ^**b**^		**Genotype counts (11/12/22)**		**Minor allele (1) freq.**			**Allelic test** ^**d**^	
**SNP rs** ^**a**^	**1**	**2**	**Cases**	**Controls**	**Cases**	**Controls**	**OR (95% CI)** ^**c**^	***P***_***asym***_	***P***_***emp***_
rs3808850 (S1)	T	A	10/53/65	66/230/174	0.2852	0.3851	0.61 (0.45-0.84)	0.0022	0.0213
rs7849191 (S2)	T	C	8/40/80	26/233/170	0.2188	0.3904	0.43 (0.30-0.60)	6.35 × 10^−7^	4.00 × 10^−5^
rs7046736 (S3)	A	C	40/69/19	65/225/180	0.5820	0.3777	2.53 (1.85-3.46)	5.92 × 10^−9^	2.00 × 10^−5^
rs2149555 (S4)	T	C	20/85/23	41/194/235	0.4883	0.2936	2.51 (1.82-3.48)	2.01 × 10^−8^	2.00 × 10^−5^
rs1536798 (S5)	A	C	28/57/43	59/228/183	0.4414	0.3681	1.30 (0.97-1.74)	0.0765	0.4500
rs10815148 (S6)	A	T	9/59/60	20/167/283	0.3008	0.2202	1.60 (1.15-2.23)	0.0057	0.0509
rs2149556 (S7)	C	T	8/63/57	86/245/139	0.3086	0.4436	0.51 (0.37-0.71)	5.23 × 10^−5^	0.0005
rs12342421 (S8)	C	G	52/54/22	43/197/230	0.6172	0.3011	3.55 (2.59-4.87)	3.76 × 10^−15^	2.00 × 10^−5^
rs10974944 (S9)	G	C	29/76/23	40/198/232	0.5234	0.2957	2.87 (2.08-3.96)	1.50 × 10^−10^	2.00 × 10^−5^
rs10119004 (S10)	G	A	10/71/47	121/248/101	0.3555	0.5213	0.46 (0.33-0.63)	1.65 × 10^−6^	4.00 × 10^−5^
rs10974947 (S11)	A	G	1/31/96	15/129/326	0.1289	0.1691	0.73 (0.48-1.11)	0.1414	0.6676
rs12343867 (S12)	C	T	22/80/23	39/186/245	0.4844	0.2809	2.60 (1.89-3.58)	3.80 × 10^−9^	2.00 × 10^−5^
rs12340895 (S13)	G	C	40/65/23	41/200/229	0.5664	0.3000	3.27 (2.37-4.51)	4.68 × 10^−13^	2.00 × 10^−5^
rs12343065 (S14)	T	C	28/77/23	41/201/228	0.5195	0.3011	2.80 (2.03-3.87)	3.80 × 10^−10^	2.00 × 10^−5^
rs7857730 (S15)	G	T	10/61/57	89/245/136	0.3164	0.4500	0.53 (0.38-0.73)	0.0001	0.0012
rs3824432 (S16)	A	G	1/37/90	26/148/296	0.1523	0.2128	0.67 (0.45-0.98)	0.0382	0.2711
rs7847294 (S17)	A	C	2/55/71	63/240/167	0.2305	0.3894	0.39 (0.27-0.56)	3.74 × 10^−7^	2.00 × 10^−5^
rs3780378 (S18)	C	T	8/58/62	84/239/147	0.2891	0.4330	0.49 (0.36-0.68)	2.25 × 10^−5^	0.0002
rs10815162 (S19)	C	G	2/43/83	40/182/248	0.1836	0.2787	0.59 (0.41-0.84)	0.0037	0.0336

Abbreviations: SNP, single nucleotide polymorphism; OR, odds ratio; *P*_*asym*_, asymptotic *P* value; *P*_*emp*_, empirical *P* value.

^a^The SNPs are listed in sequential order from the 5’ end to the 3’ end of the sense strand of the *JAK2* gene. They are also designated S1 to S19 for the sake of easy reference and discussion. Fifteen SNPs (all except S5, S6, S11 and S16) are associated with *V617F*-positive MPNs. Of these 15 MPN-associated SNPs, 7 have been reported previously (S1, S2, S3, S8, S9, S12 and S13) and 8 are novel and have not been reported previously (S4, S7, S10, S14, S15, S17, S18 and S19).

^b^Alleles 1 and 2 represent the minor and major alleles of that SNP respectively. There are 128 cases and 470 controls.

^c^Calculated for minor allele (allele 1) with major allele (allele 2) as the reference allele.

^d^Allele frequencies were compared by logistic regression with adjustment for sex and age to give the *P*_*asym*_ value. Multiple comparisons were corrected by 50,000 permutations to give the *P*_*emp*_ value.

The results agreed with the findings of Caucasian studies: the minor alleles of the *JAK2* risk-haplotype-tagging SNPs (allele *G* of rs12340895 (S13), allele *G* of rs10974944 (S9) and allele *C* of rs12343867 (S12)) were strongly associated with *V617F*-positive MPNs with descending odds ratios (ORs; 3.27, 2.87, and 2.60, respectively, with *P*_*asym*_ ≤ 3.80 × 10^−9^). All 3 SNPs statistically survived the 50,000 permutations with *P*_*emp*_ = 2.00 × 10^−5^, which is the lowest *P*_*emp*_ value achievable with 50,000 permutations. These results suggested that S9, S12, and S13 were strongly associated with *V617F*-positive MPNs. Intriguingly, we identified rs12342421 (S8) as the most significantly MPN-associated SNP (*P*_*asym*_ = 3.76 × 10^−15^ and *P*_*emp*_ = 2.00 × 10^−5^, Table 1) among the 19 SNPs in Hong Kong Chinese population. The corresponding OR for the minor allele *C* was 3.55 (95% CI, 2.59-4.87).

Given the significant difference between *V617F*-positive MPNs and healthy controls, we then examined *V617F*-negative MPN patients for the same 19 SNPs. Overall, comparison of *V617F*-negative MPNs and controls did not produce any significant association (*P*_*emp*_ >0.05) after 50,000 permutations with rs12342421 (S8) still being the strongest SNP (*P*_*emp*_ = 0.0621) (Additional file 1: Table S1). Likewise, haplotype analysis of *V617F*-negative MPNs did not yield any significant results either (*P*_*emp*_ ≥ 0.2298; data not shown). Nonetheless, a comparison of the SNP allele frequencies between *V617F*-positive and *V617F*-negative patients also did not reveal any significant difference except for rs12342421 (S8; *P*_*asym*_ = 0.0031 and *P*_*emp*_ = 0.0303) and rs12340895 (S13; *P*_*asym*_ = 0.0075 and *P*_*emp*_ = 0.0380).

We then performed haplotype analysis by comparing *V617F*-positive MPNs and controls with adjustment for sex and age. Exhaustive variable-sized sliding-window haplotype analysis was done on the 19 genotyped SNPs. PLINK [25] examined 190 windows with 1 to 19 SNPs per window, and identified 184 haplotype windows (96.8%) showing significant differences (*P*_*emp*_ < 0.05) in frequencies between patients and controls even after 50,000 permutations (Table 2). Of all the sliding haplotype windows of a given size, the haplotype window with the most significant omnibus test is shown in the third column from the right of Table 2. We examined such most significant haplotype windows for all possible window sizes, and noted that all these most significant haplotype windows *always* included rs12342421 (S8) as a constituent SNP. Of all these most significant haplotype windows, the 1-SNP window rs12342421 (S8) itself achieved the strongest association with *V617F*-positive MPNs (*P*_*asym*_ = 3.76 × 10^−15^ and *P*_*emp*_ = 2.00 × 10^−5^) (Table 2). These results were comparable to those (data not shown) based on haplotype blocks generated from Haploview (Figure 1).

**Table 2:** **Exhaustive haplotype analyses for variable-sized sliding windows across 19 genotyped** ***JAK2*** **SNPs for** ***V617F*** **-positive MPNs** ^a^

		**SW with Omnibus Test** ***P***_***emp***_ **< .05**			**Most Significant Omnibus Test**		
**SNPs, No.**	**SWs, No.**	**SWs, No.**	**First SW**	**Last SW**	**SW**	***P***_***asym***_	***P***_***emp***_
1	19	14 ^b^	S1	S18	S8 ^d^	3.76 × 10^−15^	2.00 × 10^−5^
2	18	17 ^c^	S1…S2	S18…S19	S8…S9	2.13 × 10^−14^	2.00 × 10^−5^
3	17	17	S1…S3	S17…S19	S8…S10	6.33 × 10^−14^	2.00 × 10^−5^
4	16	16	S1…S4	S16…S19	S7…S10	8.25 × 10^−13^	2.00 × 10^−5^
5	15	15	S1…S5	S15…S19	S8…S12	4.45 × 10^−12^	2.00 × 10^−5^
6	14	14	S1…S6	S14…S19	S8…S13	2.75 × 10^−12^	2.00 × 10^−5^
7	13	13	S1…S7	S13…S19	S8…S14	2.21 × 10^−12^	2.00 × 10^−5^
8	12	12	S1…S8	S12…S19	S8…S15	9.00 × 10^−12^	2.00 × 10^−5^
9	11	11	S1…S9	S11…S19	S6…S14	2.38 × 10^−11^	2.00 × 10^−5^
10	10	10	S1…S10	S10…S19	S6…S15	9.09 × 10^−12^	2.00 × 10^−5^
11	9	9	S1…S11	S9…S19	S6…S16	3.03 × 10^−11^	2.00 × 10^−5^
12	8	8	S1…S12	S8…S19	S6…S17	6.69 × 10^−11^	2.00 × 10^−5^
13	7	7	S1…S13	S7…S19	S6…S18	1.21 × 10^−10^	2.00 × 10^−5^
14	6	6	S1…S14	S6…S19	S6…S19	2.60 × 10^−10^	2.00 × 10^−5^
15	5	5	S1…S15	S5…S19	S4…S18	3.02 × 10^−9^	2.00 × 10^−5^
16	4	4	S1…S16	S4…S19	S4…S19	1.97 × 10^−9^	2.00 × 10^−5^
17	3	3	S1…S17	S3…S19	S3…S19	2.74 × 10^−9^	2.00 × 10^−5^
18	2	2	S1…S18	S2…S19	S2…S19	2.96 × 10^−8^	2.00 × 10^−5^
19	1	1	S1…S19	S1…S19	S1…S19	6.72 × 10^−8^	2.00 × 10^−5^

Abbreviations: SNP, single nucleotide polymorphism; SW, sliding window; *P*_*asym*_, asymptotic *P* value; *P*_*emp*_, empirical *P* value.

^a^The SW is shown as Sx…Sy, where Sx is the first SNP and Sy is the last SNP of the SW for *JAK2* gene. Please refer to Table 1 for the identity of the SNP concerned. Each sliding window was tested by an omnibus test adjusted for sex and age (implemented in PLINK). Multiple comparisons were corrected by running 50,000 permutations to give the *P*_*emp*_ value. The smallest *P*_*emp*_ value generated after permutation is the same for all fixed-size SWs (2 × 10^−5^); note that the lowest *P*_*emp*_ value achievable with 50,000 permutations is 2 × 10^−5^. The most significant results for each fixed-size SW are shown in the three rightmost columns. Note that, among all the 190 SWs tested, S8 always appears in the most significant SW.

^b^Of the nineteen SNPs tested, five (S5, S6, S11, S16, and S19) did not give *P*_*emp*_ < 0.05.

^c^ All the SWs gives *P*_*emp*_ < 0.05 except S5…S6.

^d^Of all the 190 SWs tested, S8 (i.e. rs12342421) alone gives the most significant result for association with *V617F*-positive MPNs.

In the 1000 Genomes Project, rs12342421 (S8) is in perfect LD (r^2^ = 1; Additional file 2: Figure S1A) with *JAK2* risk-haplotype-tagging SNPs (rs10974944, rs12343867 and rs12340895, i.e. S9, S12 and S13) for Han Chinese in Beijing (CHB), and in very strong LD (r^2^ ≥ 0.94; Additional file 2: Figure S1B) with these three SNPs in Caucasians of European ancestry (CEU). All LD plots were constructed based on solid spine of linkage disequilibrium (SSLD). The LD was moderately strong (r^2^ ≥ 0.76; Figure 1) for the corresponding pairs of SNPs in our study cohort of 128 *V617F*-positive MPN cases and 470 controls. We found that rs12342421 (S8) was not in the same LD block with *JAK2* risk-haplotype-tagging SNPs in the CEU population (Additional file 2: Figure S1B). When we further divided the sample groups and constructed LD plots, we found that the LD patterns, in descending order of LD strength (from the most correlated to the least correlated), were: the controls only ≈ the combined group of *V617F*-negative MPNs and controls (Additional file 3: Figures S2 and S3, respectively), the combined group of all MPNs and controls (Additional file 3: Figure S4), the combined group of *V617F*-positive MPNs and controls (Figure 1), all MPN cases only (Additional file 3: Figure S5), and the *V617F*-positive MPN cases only (Additional file 3: Figure S6). Overall, a higher degree of correlation was observed *among these few SNP pairs* in the 1000 Genomes Project data of CHB and CEU populations (Additional file 2: Figure S1A, B) and in our controls (Additional file 3: Figure S2) when compared with our *V617F*-positive MPN cases (Additional file 3: Figure S6).

### Genetic association of genotyped and imputed SNPs

With these significant findings, we further performed imputation for 76 additional SNPs (selected using Tagger with minor allele frequency or MAF of 0.01) with Beagle to examine the 148.7-kb region encompassing the *JAK2* locus. Manual quality control check on Beagle indicated an accuracy of >95% in imputing the missing (removed) genotypes. Consistent trends were identified when all 95 SNPs (19 directly genotyped and 76 imputed) were analysed together by logistic regression adjusted for sex and age: single-marker analysis generated the strongest association signal for rs12342421 (S8) as in our initial study. Of these 95 SNPs, 67 showed association exceeding the significance of 8 × 10^−8^ (*P*_*asym*_). The strongest association was detected for rs12342421 (S8; *P*_*asym*_ = 3.76 × 10^−15^, *P*_*emp*_ = 2.00 × 10^−5^ and OR = 3.55) while SNPs in high LD with S8 showed similar levels of association (see Table 3 for the top 20 SNPs).

**Table 3:** **Logistic regression tests: Top 20 SNPs among 95 genotyped/imputed** ***JAK2*** **SNPs in** ***V617F*** **-positive MPNs**

	**Alleles** ^**b**^		**Minor Allele Freq.**			**Allelic Test** ^**d**^	
**SNP** ^**a**^	**1**	**2**	**Cases**	**Controls**	**OR (95% CI)** ^**c**^	***P***_***asym***_	***P***_***emp***_
rs12342421 (S8)^e^	C	G	0.6172	0.3011	3.55 (2.59-4.87)	3.76 × 10^−15^	2.00 × 10^−5^
rs12347727	G	A	0.5508	0.2734	3.33 (2.43-4.56)	7.72 × 10^−14^	2.00 × 10^−5^
rs2225125	G	A	0.5508	0.2755	3.30 (2.41-4.53)	1.18 × 10^−13^	2.00 × 10^−5^
rs1327494	G	A	0.5508	0.2755	3.30 (2.41-4.53)	1.18 × 10^−13^	2.00 × 10^−5^
rs11794778	T	G	0.5508	0.2798	3.26 (2.38-4.47)	2.38 × 10^−13^	2.00 × 10^−5^
rs12340895 (S13)^e^	G	C	0.5664	0.3000	3.27 (2.37-4.51)	4.68 × 10^−13^	2.00 × 10^−5^
rs10974914	A	G	0.5547	0.3032	3.18 (2.30-4.40)	2.46 × 10^−12^	2.00 × 10^−5^
rs10974916	A	G	0.5547	0.3043	3.17 (2.29-4.38)	2.94 × 10^−12^	2.00 × 10^−5^
rs2183137	G	A	0.5508	0.2989	3.11 (2.26-4.28)	3.02 × 10^−12^	2.00 × 10^−5^
rs7851556	T	C	0.5547	0.3064	3.14 (2.27-4.34)	4.79 × 10^−12^	2.00 × 10^−5^
rs7043489	C	A	0.5547	0.3064	3.14 (2.27-4.34)	4.79 × 10^−12^	2.00 × 10^−5^
rs11794708	A	G	0.4258	0.1702	2.67 (2.02-3.53)	5.21 × 10^−12^	2.00 × 10^−5^
rs10974921	A	T	0.4258	0.1702	2.67 (2.02-3.53)	5.21 × 10^−12^	2.00 × 10^−5^
rs7030260	A	C	0.5547	0.3202	3.05 (2.20-4.23)	2.00 × 10^−11^	2.00 × 10^−5^
rs10974922	T	C	0.5547	0.3213	3.04 (2.20-4.22)	2.25 × 10^−11^	2.00 × 10^−5^
rs12349785	C	G	0.5195	0.2830	2.95 (2.14-4.06)	3.35 × 10^−11^	2.00 × 10^−5^
rs966871	T	A	0.5391	0.3021	2.86 (2.09-3.92)	5.80 × 10^−11^	2.00 × 10^−5^
rs3824433	T	C	0.5312	0.3000	2.88 (2.09-3.95)	7.73 × 10^−11^	2.00 × 10^−5^
rs1159782	C	T	0.5195	0.2936	2.89 (2.09-3.99)	1.16 × 10^−10^	2.00 × 10^−5^
rs10974944 (S9)^e^	G	C	0.5234	0.2957	2.87 (2.08-3.96)	1.50 × 10^−10^	2.00 × 10^−5^

Abbreviations: SNP, single nucleotide polymorphism; OR, odds ratio; *P*_*asym*_, asymptotic *P* value; *P*_*emp*_, empirical *P* value.

^a^The SNPs are listed in ascending order in terms of their *P*_*asym*_ among the top 20 most significantly associated *JAK2* SNPs in *V617F*-positive MPN patients. Association was tested by logistic regression with adjustment for sex and age.

^b^Alleles 1 and 2 represent the minor and major alleles of that SNP respectively. There are 128 cases and 470 controls.

^c^Calculated for minor allele (allele 1) with major allele (allele 2) as the reference allele.

^d^Allele frequencies were calculated by logistic regression with sex and age as covariates to give the *P*_*asym*_ value. Multiple comparisons were corrected by 50,000 permutations to give the *P*_*emp*_ value.

^e^These three SNPs (S8, S9 and S13) were directly genotyped in this study while the rest were imputed by Beagle v3.2 [41].

**Figure 2:**
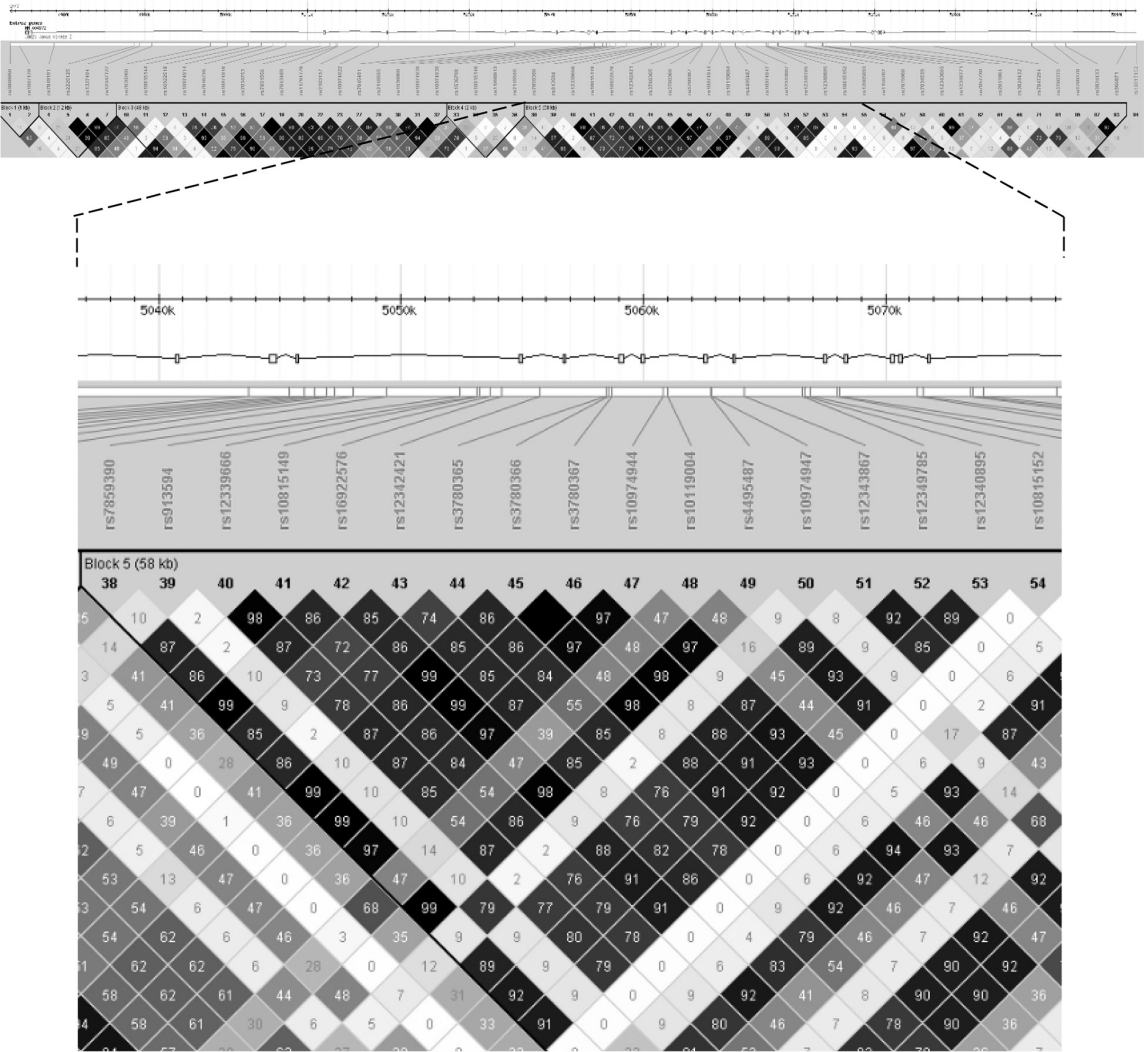
**Linkage disequilibrium pattern for 95** ***JAK2*** **SNPs for** ***V617F*** **-positive MPN cases and healthy controls.** Linkage disequilibrium plots were generated utilising the Haploview software. The values in the boxes indicate the r^2^ values between the respective pairs of SNPs and the empty boxes represent those with r^2^ = 1.0. Haplotype blocks were defined by solid spine of linkage disequilibrium.

To have an overall picture, we examined the LD structure (Figure 2) for all 95 SNPs (19 directly genotyped and 76 imputed). We realised that rs12342421 (SNP no. 43 in Figure 2) also tagged (r^2^ = 0.85) rs4495487 (SNP no. 49 in Figure 2) that was reported to be the additional variant contributing to MPN predisposition in Japanese population [14]. All the SNPs within this haplotype block showed very strong extent of LD (r^2^ close to 1; bottom panel of Figure 2).

Likewise, exhaustive haplotype analysis was performed on these 95 SNPs to further restrict the linked region and identify the most probable MPN-predisposing variants or haplotypes (Additional file 4: Table S2). Age and sex were adjusted as covariates. The SNP rs12342421 (S8) again topped the 1634 haplotype windows as a 1-SNP window (S8 itself): *P*_*asym*_ = 3.76 × 10^−15^ and *P*_*emp*_ = 2.00 × 10^−5^ for 50,000 permutations (Additional file 4: Table S2). Adjacent SNPs spanning across rs12342421 formed the most significantly associated haplotypes among the rest as in the sliding windows for the 19 directly genotyped SNPs. The SNP rs12342421 (S8) was obviously important because almost all the statistically significant haplotypes carried this SNP.

### Conditional logistic regression

Based on the results from PLINK, we tested the individual effect on disease association of the strongest MPN-associated SNP (rs12342421, i.e. S8) and the risk-haplotype-tagging SNPs (rs10974944, rs12343867 and rs12340895, i.e. S9, S12 and S13) in the corresponding sliding window. The shortest and most significant sliding haplotype window containing these four SNPs was the 6-SNP window S8…S13 (*P*_*asym*_ = 2.75 × 10^−12^; Table 2), which was therefore selected for conditional logistic regression analysis. Conditional analysis for the independent effect of one SNP at a time suggested that *only* rs12342421 (S8) contributed an independent effect to the significant association between the 6-SNP window and *V617F*-positive MPN cases (*P* = 0.0005 for omnibus test of independent effect, Table 4). Logically, controlling for all the single SNPs except rs12342421 (S8) yielded a reduced but still statistically significant *P* value of ≤0.0072 while controlling for rs12342421 (S8) demolished the significance (*P* = 0.4360) (Table 4). In other words, we could not detect any significant association when rs12342421 (S8) was removed from the combination, and the original risk-haplotype-tagging SNPs (S9, S12 and S13) did not explain all the association signals.

**Table 4:** **Conditional haplotype-based test: independent effects of individual** ***JAK2*** **SNPs on the 6-SNP sliding window S8…S13** ^a^

	**Conditional haplotype-based association test,** ***P*** **value**	
**Sx** ^**b**^	**Independent effect of Sx** ^**c**^	**Controlling for Sx** ^**d**^
rs12342421 (S8)	0.0005	0.4360
rs10974944 (S9)	– ^e^	0.0072
rs10119004 (S10)	0.4700	2.84 × 10^−7^
rs10974947 (S11)	0.2480	1.79 × 10^−14^
rs12343867 (S12)	0.7970	0.0019
rs12340895 (S13)	– ^e^	0.0072

^a^This table shows the individual effects of the constituent single nucleotide polymorphisms (SNPs) on the shortest and most significant sliding window that contains the most impressive SNP in our study (rs12342421, i.e. S8) and the risk-haplotype-tagging SNPs (rs10974944, rs12343867 and rs12340895, i.e. S9, S12 and S13). Conditional logistic regression was performed with adjustment for sex and age. The shortest and most significant sliding window carrying these four SNPs is S8…S13 (see Table 2). The conditional omnibus test invoked by the “--chap” command of PLINK gives a *P* value of 1.34 × 10^−14^ (based on likelihood ratio test).

Note that this *P* value is similar, but not identical, to the *P* value of 2.75 × 10^−12^ (based on Wald test, Table 2) generated by the omnibus test of logistic regression invoked by the “--logistic” command of PLINK in the sliding-window approach.

^b^Sx indicates the SNP tested for an independent effect one at a time by the conditional haplotype-based analysis of the sliding window S8…S13. Please refer to Table 1 for the identity of the SNPs concerned.

^c^Omnibus *P* value for the effect of Sx that is independent of the other SNPs in the sliding window S8…S13.

^d^Omnibus *P* value for the sliding window S8…S13 when Sx is controlled for.

^e^Not a valid comparison due to identical alternate and null models

Our data suggested that *JAK2* germline polymorphisms, especially rs12342421 (S8), were significantly associated with *V617F*-positive MPN in Hong Kong Chinese population.

## Discussion

There has been evidence suggesting that *JAK2* 46/1 haplotype contributed to the development of *V617F-*positive MPNs, but the findings for *V617F-*negative MPNs are inconsistent and less convincing. While most of the studies detected no association between the risk-haplotype and *V617F*-negative MPNs [9,10,17,20-22], significant association with *V617F*-negative MPN patients was reported in two studies with bigger sample size (n = 108 and 53) [12,13]. In the light of recent Chinese studies that the *JAK2* haplotype poses a higher risk of developing *V617F*-positive MPNs [19,20], we employed a case–control study design to explore the described genetic susceptibility to MPNs in the Hong Kong Chinese population. To avoid missing any potential causal variant in the region, we investigated not only the risk-haplotype-tagging SNPs but also a total of 95 SNPs in two stages with an increased sample size. In the first stage, we genotyped 19 tag SNPs of the *JAK2* locus. In the second stage, we carried out genotype imputation on additional 76 *JAK2* SNPs. We then combined the 19 directly genotyped SNPs and the 76 imputed SNPs (95 in total), and carefully examined both datasets by both single-marker and haplotype analyses.

After single-marker analysis, we adopted a variable-sized sliding-window strategy to examine haplotypic effects in an unbiased manner. This exhaustive approach is best suited for capturing the haplotypes of all possible sliding-widow sizes (including single markers) that are most significantly associated with MPNs [26]. This comprehensive approach identified from the 19 directly genotyped SNPs 184 haplotype windows that showed significant association (~97% of all 190 haplotype windows; *P*_*emp*_ < 0.05, Table 2) even after correction for multiple comparisons. However, single-marker analyses of both the 19 SNPs and the 76 imputed SNPs showed that *V617F*-positive MPNs were associated more significantly with the single SNP rs12342421 (S8, also tagging the risk haplotype) than the haplotypes (Table 1 vs Table 2, and Table 3 vs Additional file 4: Table S2)) although strong association between the risk-haplotype-tagging SNPs (rs10974944, rs12343867 and rs12340895, i.e., S9, S12 and S13) and *V617F*-positive MPNs was also evident. The *C* allele rs12342421 (S8) was enriched in *V617F*-positive MPN patients when compared with controls. Our conditional logistic regression further demonstrated that this single SNP contributed an *independent* effect to the most significant association between haplotypes and MPNs (Table 4) – a novel finding not previously reported. Analysis showed that rs12342421 (S8) had stronger association when it was *not* combined with other SNPs, i.e. as a single marker (Table 2). This means that the effect of rs12342421 (S8) became less significant when it was combined with other SNPs. The results also imply that the original risk-haplotype-tagging SNPs (S9, S12 and S13) do not explain all the association signals; this finding is intriguing because many studies only focused on one or more of these three risk-haplotype-tagging SNPs.

Although rs12342421 (S8) was analysed in an early study, the results were never reported explicitly [10]. Two other studies indeed reported the association of rs12342421 (S8) with MPNs in Caucasians [16,27]. However, both studies did *not* investigate whether rs12342421 (S8) contributed an effect independent of the *JAK2* 46/1 haplotype [16,27]. In addition, Pardanani *et al*. [16] is so far the *only* study that reported *opposite* effects (high-risk vs protective) for PV and ET for SNPs found to be associated with these MPN subtypes. Zerjavic *et al*. [27] is so far also the *only* study that failed to demonstrate association between MPNs and rs12343867 (S12) – the SNP most commonly used for tagging the 46/1 haplotype, while other risk-haplotype tagging SNPs still showed association with MPNs. Zerjavic *et al*. [27] also reported a less significant association for rs12342421 (S8) than for rs10974944 (S9) – a finding different from ours (Table 1).

Overall, 19 tag SNPs were genotyped in this study and 15 found to be associated with *V617F*-postive MPNs (see footnote a of Table 1). Of these, 7 have been previously reported to be associated with MPNs in one or more studies [9-23], including the most three commonly studied risk-haplotype-tagging SNPs rs10974944, rs12343867 and rs12340895 (i.e. S9, S12 and S13). The remaining eight SNPs are *novel* MPN-associated SNPs and have not been reported previously. In contrast, four SNPs that have been reported to be associated MPN or its subtypes were *not* genotyped experimentally or by imputation in the current study: rs10758677 in a European study [9], rs10758669 in an American study [16], rs11999802 in another American study [18] and rs10118930 in a Chinese study [21]. Of particular interest is rs11999802, a genome-wide significant SNP (*P* = 1.8 × 10^−8^) associated with PV with an allelic OR of 4.41 in a small-scale genome-wide association study involving 34 PV patients and 3,278 control subjects of European ancestry [18].

Our results show that the significant association between *JAK2* polymorphisms and MPNs in Hong Kong Chinese is comparable to the results in other populations. However, we found that rs12342421 (S8) was *not* in the same LD block with *JAK2* risk-haplotype-tagging SNPs in the CEU population (Additional file 2: Figure S1B) although it is still in strong LD (r^2^ close to 1) with *JAK2* risk-haplotype-tagging SNPs. This may explain why rs12342421 (S8), rather than the *JAK2* risk-haplotype-tagging SNPs, exhibits a stronger association with MPNs in our population. When we examined the effect of *V617F* on the extent of LD, we found that the r^2^ between rs12342421 (S8) and other *JAK2* risk-haplotype-tagging SNPs decreased in a *V617F*-dependent manner. We observed that controls had stronger LD (r^2^) among these SNPs than cases, and that cases without *V617F* mutation had stronger LD than cases with *V617F* mutation (Additional file 3: Figures S2-S6 and Figure 1). The r^2^ values were much lower when *V617F*-positive cases were included to construct the LD plot. It has been demonstrated that there can be extensive variation in the extent of LD between cases and controls in a region of genetic association [28]. The variation in LD patterns observed in our cases (especially cases with *V617F*) and controls suggests that the region surrounding rs12342421 (S8) is associated with MPNs. While current genetic maps can be used to examine the LD structure, fine mapping at higher resolution may still be required to sufficiently examine the region because recombination occurs not only in hot spots [29].

We explored the potential biological functions of the genotyped genetic markers with several web-based SNP prediction tools that are supported by regularly updated databases and software tools: SNPnexus [30], SNP Function Prediction (FuncPred) [31], F-SNP [32] and MaInspector [33]. In silico analysis predicted no known function for rs12342421 (S8) and other genotyped SNPs except that one 5’-upstream SNP (rs3808850 (S1)) and two intronic SNPs (rs7849191 (S2) and rs3780378 (S18)) were predicted by FuncPred to be involved in transcription factor binding sites. Experimental functional studies may be required to clarify this issue.

We then conducted an analysis of expression quantitative trait loci (eQTL) across the *JAK2* gene (142.8 kb) with several online tools: eQTL resources @ the Pritchard lab [34], seeQTL [35], and UCSC Genome Browser [36]. This analysis did not detect any regulatory regions within the two recombination hotspots encompassing the *JAK2* gene [9].

These circumstantial findings suggest that the causal variants driving the disease development may not be the SNPs or haplotypes reported here, but some untyped variants in LD with these markers. However, it is also possible that the potential functions of the associated SNPs are some biological processes that are not well captured by current functional annotation software. Owing to limited eQTL studies on different tissues or cell types, eQTL studies might provide only limited knowledge for linking regulatory variants to specific genes in different tissues or cell types. There might be some other eQTLs that have not been curated, leading to the limited information [37].

The distribution of *V617F* in our Hong Kong MPN patients (PV, ET and PMF) is similar to those in other studies [4-7]. This justified our comparable findings to those in other populations. Taken together, our results corroborate the findings that *JAK2* variants are predisposing factors for MPN development dependent on *V617F* in Hong Kong Chinese, especially rs12342421 (S8). Conceivably, the failure to detect, in our study, the association between *V617F*-negative MPNs and controls as reported elsewhere [12,13] can be ascribed to the small sample size of the cases (n = 44). Larger sample size would probably be needed to detect an association for *V617F*-negative MPNs.

To the best of our knowledge, we are the first to perform genotype imputation in genetic association studies of MPNs. Being an essential component in genetic association study, imputation enabled us to test many untyped markers for associations with MPNs and hence increased the chance to identify causal variants. Although we failed to find the causal variant, imputation together with conditional logistic regression indeed further strengthens our confidence to conclude that rs12342421 (S8) contributed an independent effect to the most significant association between *JAK2* risk haplotype and MPNs.

## Conclusions

Fifteen *JAK2* germline polymorphisms were associated with MPN patients with *V617F* mutation in Hong Kong Chinese population. The single *JAK2* SNP rs12342421 (S8) was associated with predisposition to the development of *V617F*-positive MPN by 3.55 fold for the minor allele *C*, but independent of the *JAK2* 46/1 haplotype. No significant association was found between *V617F*-negative MPN patients and the *JAK2* risk alleles. We have presented some plausible arguments that S8 is likely to be involved in the pathogenesis of MPN. However, further functional validation is necessary to prove its involvement in the disease development.

## Methods

### Subjects and DNA samples

Participants were Hong Kong Chinese MPN patients diagnosed according to the WHO 2008 criteria [1] and recruited from six local hospitals. Every patient signed a written informed consent. Both blood and saliva samples were collected from patients. Blood DNA was extracted with FlexiGene DNA Kit (Qiagen) and used for *V617F* detection. Saliva samples were collected using the Oragene DNA self-collection kit (DNA Genotek), and saliva DNA was extracted according to the manufacturer’s instructions and used for SNP genotyping. As for controls, 470 blood samples from anonymous healthy Chinese donors were collected from the Hong Kong Red Cross Blood Transfusion Service and these donors were matched to the MPN patients for sex and age as much as possible. DNA extracted from control blood samples were used for both *V617F* detection and SNP genotyping. Assuming a prevalence of 0.00002, MAF of 0.1, genotypic relative risk of 2.5 for Aa and 5.0 for AA, we estimated that a sample size of 128 cases and 460 controls would have 80% power (Genetic Power Calculator) [38]. This study was approved by the Human Subjects Ethics Sub-Committee of the University (reference numbers: 20090801001 and 20111118001) and Research Ethics Committees of the hospitals, according to the guideline of the Declaration of Helsinki. The Research Ethics Committees of the hospitals under Hospital Authority included the following: Kowloon West Cluster Clinical Research Ethics Committee (reference number: KW/EX/09-076); Research Ethics Committee, Kowloon Central/Kowloon East Clusters (KC/KE-09-0120/FR-3); Joint The Chinese University of Hong Kong–New Territories East Cluster Clinical Research Ethics Committee (reference number: CRE-2009.423); and Ethics Committee, Hong Kong Easter Cluster (HKEC-2009-069). All experiments were performed in the research laboratories of Department of Health Technology and Informatics, The Hong Kong Polytechnic University.

### *JAK2V617F* mutation analysis

DNA from all blood samples of patients and controls were tested for *V617F* by amplification refractory mutation system modified from Jones *et al*. [4]. PCR products were analysed by electrophoresis on 5% polyacrylamide gels. Details are provided in Additional file 5.

### SNP selection and genotyping

First, we attempted to identify *JAK2* germline variants that are associated with the development of MPNs in our Hong Kong Chinese population in addition to the *JAK2* risk haplotypes. Tag SNPs were selected using the Tagger software from a 148.7-kb region encompassing the *JAK2* locus and its potential regulatory regions (3 kb upstream and downstream of *JAK2*) with MAF ≥0.1 and pairwise tagging algorithm, r^2^ ≥ 0.8, based on HapMap CHB database (release #24/phase I) [24]. In line with previous studies, we force-included the reported risk-haplotype-tagging SNPs (rs10974944, rs12343867, and rs12340895; i.e. S9, S12 and S13) [9-11,17]. To avoid the complication from loss of heterozygosity resulting from somatic isodisomy in clonal myeloid cells, DNA from patients’ saliva samples (instead of blood samples) was used for SNP genotyping. In this study, two methods were used for genotyping SNPs (Additional file 5): 14 SNPs by restriction fragment length polymorphism analysis and 5 SNPs by unlabelled probe melting analysis [39-43]. Details of primer sequences and reaction conditions are given in (Additional file 6: Table S3). For illustration, the restriction fragments and the banding patterns of a SNP are shown in (Additional file 7: Figure S7), and the melting curves of another SNP in (Additional file 8: Figure S8).

### Imputation of genotypes for 76 *JAK2* SNPs

Genotypes of 76 additional SNPs within the 148.7-kb region under study were imputed by Beagle v3.2 [44]. One of the imputed SNPs was rs4495487, which was recently reported to contribute to MPN development in the Japanese population [14]. The genotype data of the 1000 Genomes Project (phase 1) based on 97 CHB subjects were used as the reference panel. We manually performed a quality control check by removing some of the known genotypes of the 19 directly genotyped SNPs, and imputed them with Beagle v3.2. The post-imputation results were merged with the original data to check for the imputation accuracy.

### Statistical analysis

Genotypes were tested for Hardy-Weinberg equilibrium (HWE) by Fisher’s exact test using PLINK (ver.1.07) [25] prior to data analysis. PLINK was used for statistical analysis for all the 19 directly genotyped SNPs and the 76 imputed SNPs, and also the haplotype association tests. Single-marker and haplotype analyses were conducted between cases and controls with logistic regression adjusted for sex and age (age at diagnosis for MPN patients) as covariates; the respective asymptotic *P* value was denoted as *P*_*asym*_. Correction for multiple comparisons was achieved by generating empirical *P* values (*P*_*emp*_) based on 50,000 permutations, i.e., swapping of the case–control status 50,000 times. Haplotypes were defined by a variable-sized sliding-window approach based on all possible sizes of SNPs spanning the whole genomic region. Subsequently, we studied the contribution of individual SNPs to significant haplotype association with disease by conditional logistic regression analysis. Haploview v4.2 [45] was used to generate the linkage disequilibrium (LD) map of the *JAK2* gene based on an algorithm called solid spine of linkage disequilibrium (SSLD) [45].

## Additional files

Additional file 1: Table S1. Allelic association tests for 19 genotyped SNPs of the *JAK2* gene in *V617F*-negative MPNs. Additional file 2: Figure S1. Haploview-generated linkage disequilibrium (LD) map of 19 *JAK2* SNPs in Han Chinese in Beijing (A) and Caucasians of European ancestry (B) based on the 1000 Genomes Project data. LD plots were generated utilising the Haploview software. The values in the boxes indicate the r^2^ values between the respective pairs of SNPs and the empty boxes represent those with r^2^ = 1.0. Haplotype blocks are defined by solid spine of linkage disequilibrium. Additional file 3: Figure S2. Haploview-generated linkage disequilibrium map of 19 SNPs in the *JAK2* gene for only the 470 controls of our study. **Figure S3.** Haploview-generated linkage disequilibrium map of the 19 SNPs in the *JAK2* gene for 44 *V617F*-negative MPN cases and 470 controls of our study. **Figure S4.** Haploview-generated linkage disequilibrium map of the 19 SNPs in the *JAK2* gene for all the 172 *V617F*-positive and -negative MPNs patients and the 470 controls of our study. **Figure S5.** Haploview-generated linkage disequilibrium map of the 19 SNPs in the *JAK2* gene for the 172 *V617F*-positive and -negative MPNs patients of our study. **Figure S6.** Haploview-generated linkage disequilibrium map of the 19 SNPs in the *JAK2* gene for the 128 *V617F*-positive MPNs patients of our study. Additional file 4: Table S2. Summary of exhaustive haplotype analyses based on age- and sex-adjusted omnibus tests for sliding windows of up to 19 SNPs per window for 95 genotyped/imputed *JAK2* SNPs for *V617F*-positive MPNs. Additional file 5:
**Genotyping of** ***JAK2*** **mutation and single nucleotide polymorphisms (SNPs).**
Additional file 6: Table S3.
*JAK2* SNPs: Genotyping methods, primers, probes and key reaction conditions for PCR. Additional file 7: Figure S7. SNP genotyping by restriction fragment length polymorphism. For illustration, rs10119004 (S10) is used as an example. A PCR fragment of 244 bp (see Additional file; **Table S3**) is amplified to encompass the SNP site. Upper panel: Restriction patterns for the two alleles of rs10119004 (S10) upon restriction digestion by the restriction enzyme HphI. Lower panel: Electrophoresis banding patterns on 8% polyacrylamide gel and stained with SYBR Green I. The DNA ladder is the 1 kb Plus DNA Ladder (lane M) from Invitrogen Life Technologies. Additional file 8: Figure S8. SNP genotyping by unlabelled probe melting analysis. For illustration, rs7849191 (S2) is used as an example. A PCR fragment of 115 bp (see Additional file 6: Table S3) is amplified to encompass the SNP site. The unlabelled probe is designed to match the C allele in this example. The probe-amplicon duplex of the homozygous genotype CC has a higher melting temperature than the probe-amplicon duplex of the homozygous genotype TT. Two peaks are obtained for the heterozygous genotype CT.

## Abbreviations

SNP: Single nucleotide polymorphism
*JAK2*: Janus kinase 2
MPN: Myeloproliferative neoplasm
PV: Polycythaemia vera
ET: Essential thromocythaemia
PMF: Primary myelofibrosis
LD: Linkage disequilibrium
MAF: Minor allele frequency
OR: Odds ratio
CEU: Caucasians with European ancestry
CHB: Han Chinese in Beijing
SSLD: Solid spine of linkage disequilibrium
