# Supplementary material for: Genetic association between germline JAK2 polymorphisms and myeloproliferative neoplasms in Hong Kong Chinese population: a case–control study
Source: BMC Genet. 2014 Dec 20;15:147. doi: 10.1186/s12863-014-0147-y (PMC4293821; doi:10.1186/s12863-014-0147-y)
Supplement: Additional file 1: Table S1. — Allelic association tests for 19 genotyped SNPs of the JAK2 gene in V617F-negative MPNs. [file 12863_2014_147_MOESM1_ESM.doc]

**Additional file 1:**

**Table S1.** Allelic association tests for 19 genotyped tag SNPs of the *JAK2* gene in *V617F*-negative MPNs

|  | Alleles ^b^ | | Genotype Counts (11/12/22)^c^ | | Minor allele (1) freq. | |  | Allelic Test ^d^ | |
| --- | --- | --- | --- | --- | --- | --- | --- | --- | --- |
| SNP rs ^a^ | 1 | 2 | Cases | Controls | Cases | Controls | OR (95% CI) ^c^ | *P_asym_* | *P_emp_* |
| rs3808850 (S1) | T | A | 3/21/20 | 66/230/174 | 0.3068 | 0.3851 | 0.68 (0.42-1.11) | 0.1253 | 0.6068 |
| rs7849191 (S2) | T | C | 3/20/21 | 67/233/170 | 0.2955 | 0.3904 | 0.63 (0.39-1.04) | 0.0674 | 0.4081 |
| rs7046736 (S3) | A | C | 6/31/7 | 65/225/180 | 0.4886 | 0.3777 | 1.60 (1.01-2.53) | 0.0434 | 0.2935 |
| rs2149555 (S4) | T | C | 5/27/12 | 41/194/235 | 0.4205 | 0.2936 | 1.75 (1.11-2.75) | 0.0156 | 0.1267 |
| rs1536798 (S5) | A | C | 5/27/12 | 59/228/183 | 0.4205 | 0.3681 | 1.27 (0.80-2.01) | 0.2980 | 0.9225 |
| rs10815148 (S6) | A | T | 0/22/22 | 20/167/283 | 0.2500 | 0.2202 | 1.17 (0.69-1.98) | 0.5666 | 0.9981 |
| rs2149556 (S7) | C | T | 2/26/16 | 86/245/139 | 0.3409 | 0.4436 | 0.63 (0.39-1.01) | 0.0573 | 0.3565 |
| rs12342421 (S8) | C | G | 7/25/12 | 43/197/230 | 0.4432 | 0.3011 | 1.85 (1.18-2.90) | 0.0078 | 0.0621 |
| rs10974944 (S9) | G | C | 5/27/12 | 40/198/232 | 0.4205 | 0.2957 | 1.74 (1.10-2.76) | 0.0181 | 0.1328 |
| rs10119004 (S10) | G | A | 13/26/5 | 101/248/121 | 0.5909 | 0.4787 | 1.62 (1.02-2.57) | 0.0441 | 0.2812 |
| rs10974947 (S11) | A | G | 0/15/29 | 15/129/326 | 0.1705 | 0.1691 | 1.02 (0.57-1.83) | 0.9061 | 1.0000 |
| rs12343867 (S12) | C | T | 4/27/13 | 39/186/245 | 0.3977 | 0.2809 | 1.69 (1.07-2.66) | 0.0246 | 0.1822 |
| rs12340895 (S13) | G | C | 4/29/11 | 41/200/229 | 0.4205 | 0.3000 | 1.72 (1.09-2.74) | 0.0191 | 0.1560 |
| rs12343065 (S14) | T | C | 5/27/12 | 41/201/228 | 0.4205 | 0.3011 | 1.71 (1.08-2.70) | 0.0222 | 0.1737 |
| rs7857730 (S15) | G | T | 1/29/14 | 89/245/136 | 0.3523 | 0.4500 | 0.64 (0.40-1.03) | 0.0730 | 0.4060 |
| rs3824432 (S16) | A | G | 0/15/29 | 26/148/296 | 0.1705 | 0.2128 | 0.78 (0.44-1.38) | 0.4091 | 0.9731 |
| rs7847294 (S17) | A | C | 1/26/17 | 63/240/167 | 0.3182 | 0.3894 | 0.70 (0.43-1.15) | 0.1598 | 0.7130 |
| rs3780378 (S18) | C | T | 2/27/15 | 84/239/147 | 0.3523 | 0.4330 | 0.69 (0.43-1.11) | 0.1190 | 0.6211 |
| rs10815162 (S19) | C | G | 1/18/25 | 40/182/248 | 0.2273 | 0.2787 | 0.78 (0.47-1.31) | 0.3464 | 0.9494 |

Abbreviation: SNP, single nucleotide polymorphism; OR, odds ratio; *P_asym_*, asymptotic *P* value; *P_emp_*, empirical *P* value.

^a^ The SNPs are listed in sequential order from the 5’ end to the 3’ end of the sense strand of the *JAK2* gene. They are also designated S1 to S19 for the sake of easy reference and discussion.

^b^ Alleles 1 and 2 represent the minor and major alleles of that SNP respectively. There are 44 cases and 470 controls.

^c^ Calculated for minor allele (allele 1) with major allele (allele 2) as the reference allele.

^d^ Allele frequencies were compared between cases and controls by logistic regression adjusted for sex and age to give the *P_asym_* value. Multiple comparison was corrected by 50,000 permutations to give the *P_emp_* value.
