# Supplementary material for: Genetic association between germline JAK2 polymorphisms and myeloproliferative neoplasms in Hong Kong Chinese population: a case–control study
Source: BMC Genet. 2014 Dec 20;15:147. doi: 10.1186/s12863-014-0147-y (PMC4293821; doi:10.1186/s12863-014-0147-y)
Supplement: Additional file 2: Figure S1. — Haploview-generated linkage disequilibrium (LD) map of 19 JAK2 SNPs in Han Chinese in Beijing (A) and Caucasians of European ancestry (B) based on the 1000 Genomes Project data. LD plots were generated utilising the Haploview software. The values in the boxes indicate the r2 values between the respective pairs of SNPs and the empty boxes represent those with r2 = 1.0. Haplotype blocks are defined by solid spine of linkage disequilibrium. [file 12863_2014_147_MOESM2_ESM.doc]

**
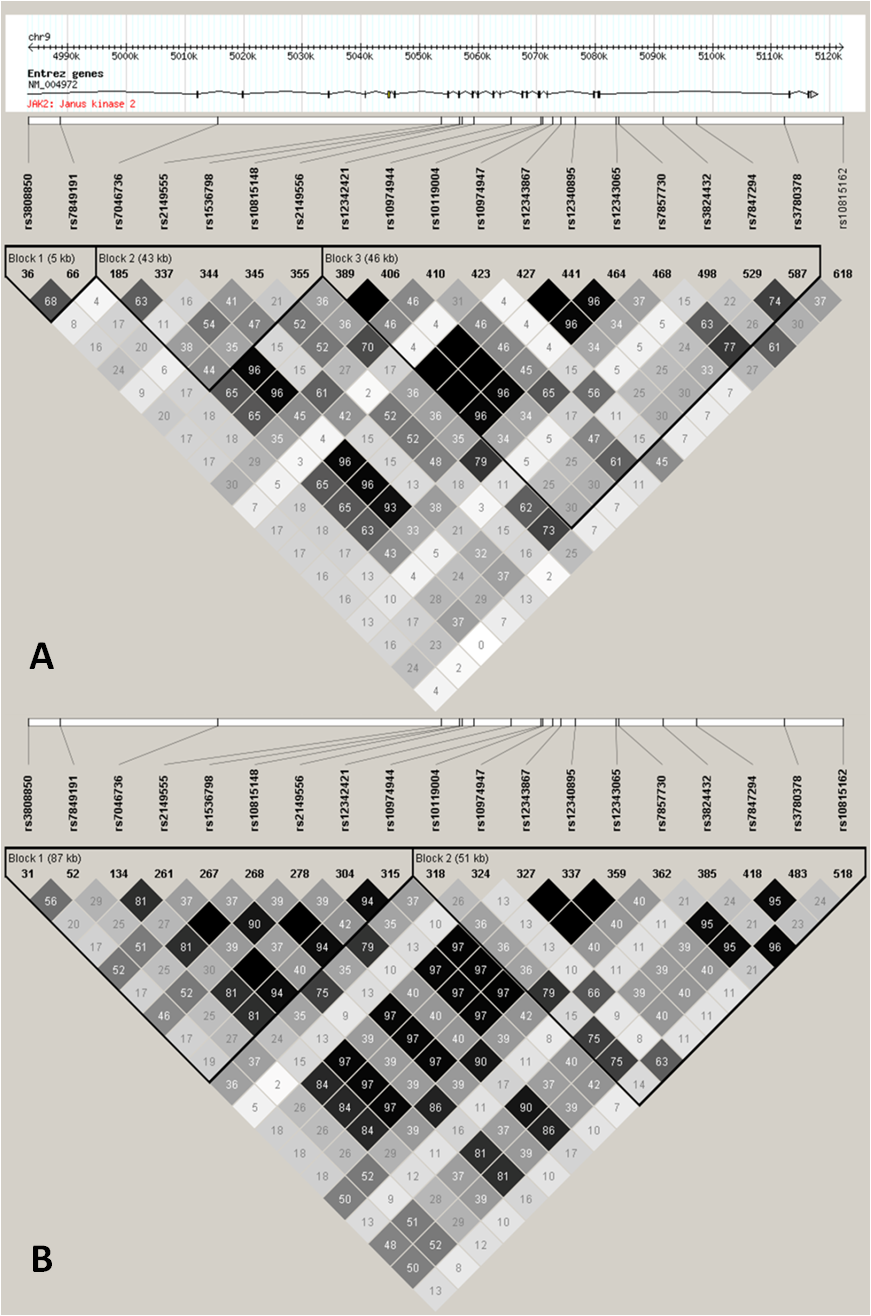
**

**Additional file 2: Fig S1.** Haploview-generated linkage disequilibrium (LD) map of 19 *JAK2* SNPs in Han Chinese in Beijing (A) and Caucasians of European ancestry (B) based on the 1000 Genomes Project data. LD plots were generated utilising the Haploview software. The values in the boxes indicate the r^2^ values between the respective pairs of SNPs and the empty boxes represent those with r^2^ = 1.0. Haplotype blocks are defined by solid spine of linkage disequilibrium.
