# Supplementary material for: Genetic association between germline JAK2 polymorphisms and myeloproliferative neoplasms in Hong Kong Chinese population: a case–control study
Source: BMC Genet. 2014 Dec 20;15:147. doi: 10.1186/s12863-014-0147-y (PMC4293821; doi:10.1186/s12863-014-0147-y)
Supplement: Additional file 3: Figure S2. — Haploview-generated linkage disequilibrium map of 19 SNPs in the JAK2 gene for only the 470 controls of our study. Figure S3. Haploview-generated linkage disequilibrium map of the 19 SNPs in the JAK2 gene for 44 V617F-negative MPN cases and 470 controls of our study. Figure S4. Haploview-generated linkage disequilibrium map of the 19 SNPs in the JAK2 gene for all the 172 V617F-positive and -negative MPNs patients and the 470 controls of our study. Figure S5. Haploview-generated linkage disequilibrium map of the 19 SNPs in the JAK2 gene for the 172 V617F-positive and -negative MPNs patients of our study. Figure S6. Haploview-generated linkage disequilibrium map of the 19 SNPs in the JAK2 gene for the 128 V617F-positive MPNs patients of our study. [file 12863_2014_147_MOESM3_ESM.doc]

**Additional file 3: Figures S2 to S6 (LD maps of various sample groups)**


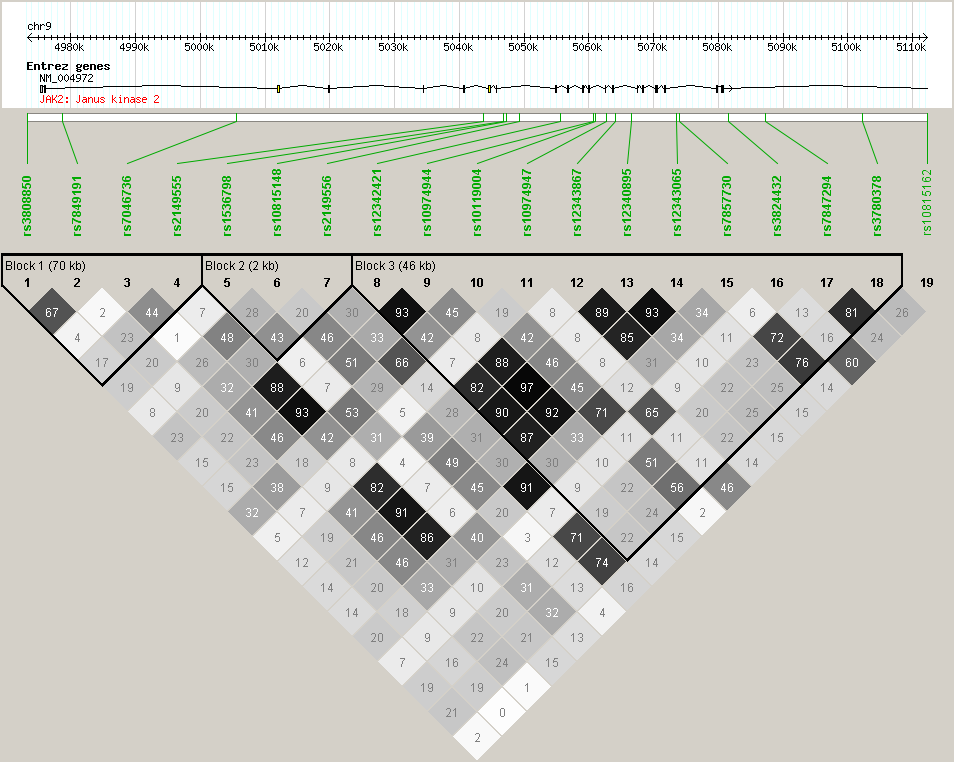


**Additional file 3: Figure S2.** Haploview-generated linkage disequilibrium (LD) map of 19 SNPs in the *JAK2* gene for only the 470 controls of our study.LD plots were generated utilising the Haploview software. The values in the boxes indicate the r^2^ values between the respective pairs of SNPs and the empty boxes represent those with r^2^ = 1.0. Haplotype blocks are defined by solid spine of linkage disequilibrium.


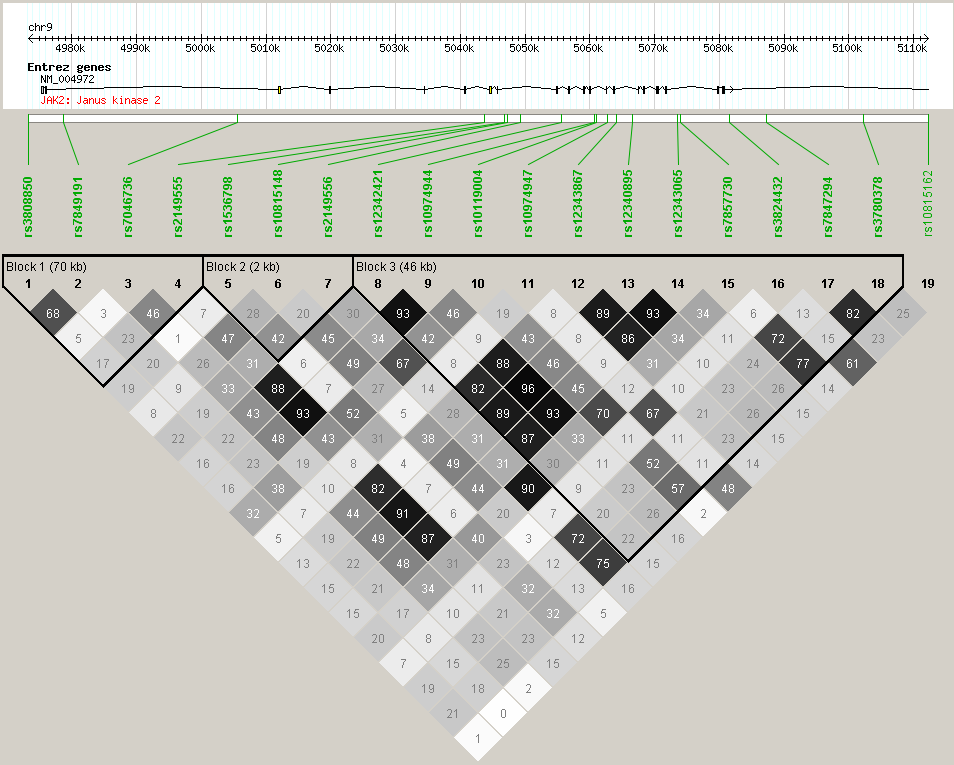


**Additional file 3: Figure S3.** Haploview-generated linkage disequilibrium (LD) map of the 19 SNPs in the *JAK2* gene for 44 *V617F*-negative MPN cases and 470 controls of our study.LD plots were generated utilising the Haploview software. The values in the boxes indicate the r^2^ values between the respective pairs of SNPs and the empty boxes represent those with r^2^ = 1.0. Haplotype blocks are defined by solid spine of linkage disequilibrium.


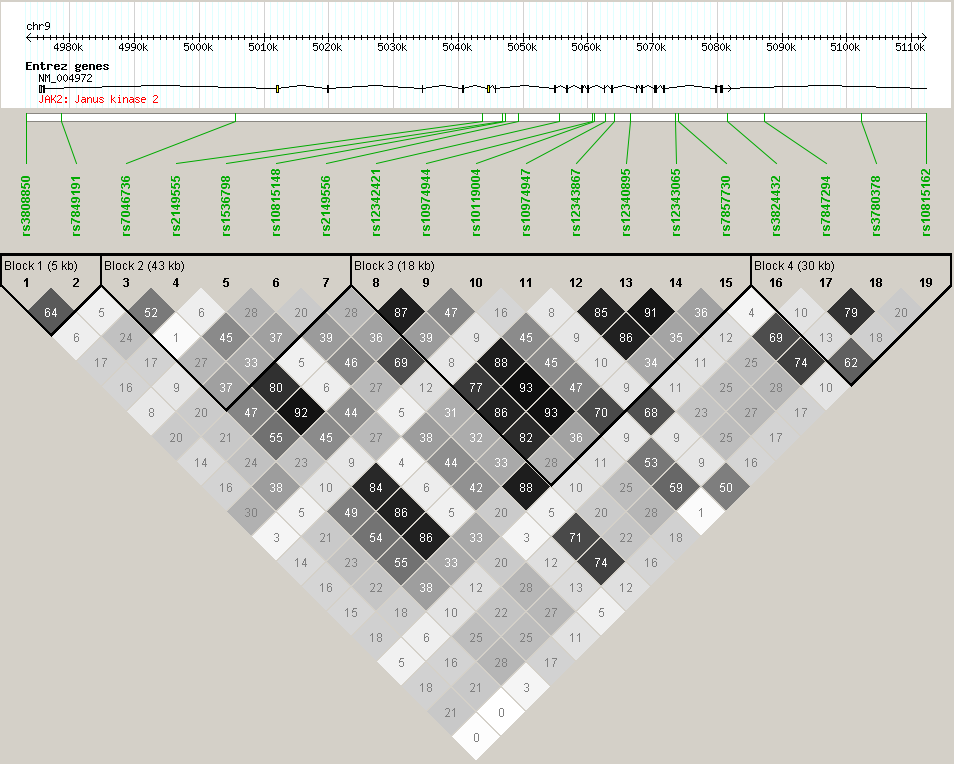


**Additional file 3: Figure S4.** Haploview-generated linkage disequilibrium (LD) map of the 19 SNPs in the *JAK2* gene for all the 172 *V617F*-positive and -negative MPNs patients and the 470 controls of our study. LD plots were generated utilising the Haploview software. The values in the boxes indicate the r^2^ values between the respective pairs of SNPs and the empty boxes represent those with r^2^ = 1.0. Haplotype blocks are defined by solid spine of linkage disequilibrium.


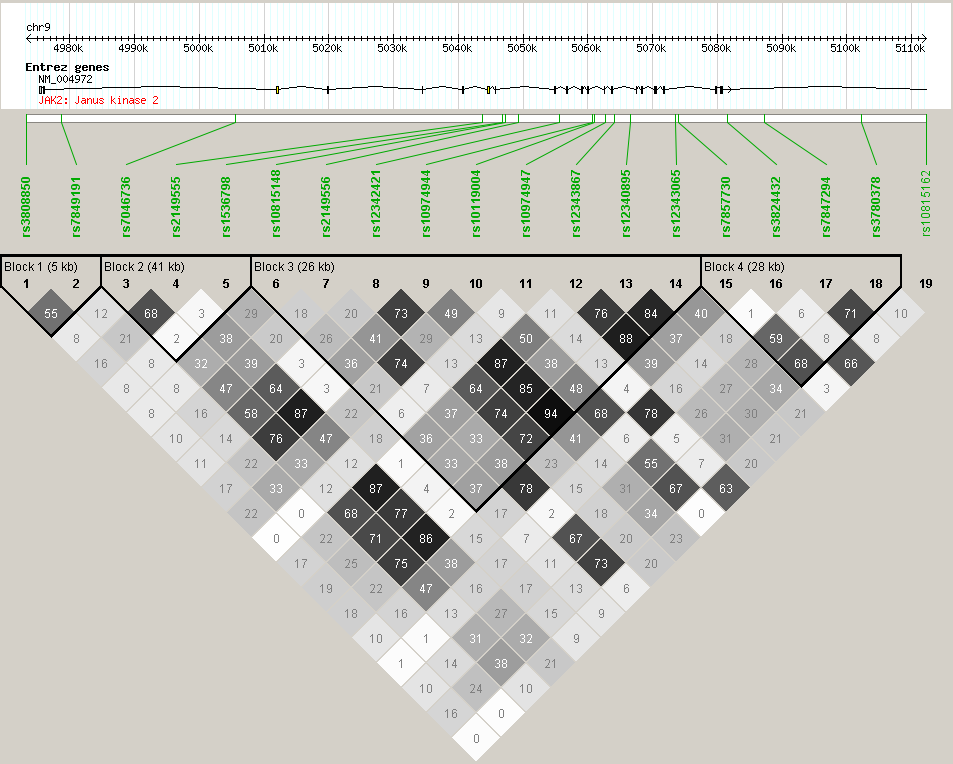


**Additional file 3: Figure S5.** Haploview-generated linkage disequilibrium (LD) map of the 19 SNPs in the *JAK2* gene for the 172 *V617F*-positive and -negative MPNs patients of our study. LD plots were generated utilising the Haploview software. The values in the boxes indicate the r^2^ values between the respective pairs of SNPs and the empty boxes represent those with r^2^ = 1.0. Haplotype blocks are defined by solid spine of linkage disequilibrium.


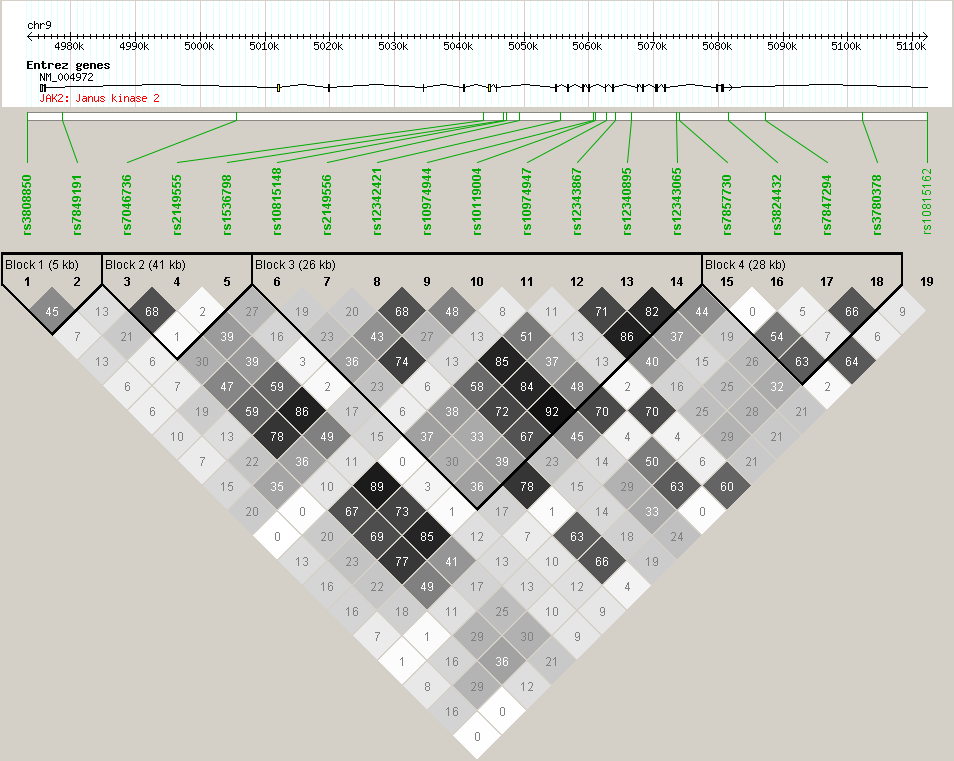


**Additional file 3: Figure S6.** Haploview-generated linkage disequilibrium (LD) map of the 19 SNPs in the *JAK2* gene for the 128 *V617F*-positive MPNs patients of our study. LD plots were generated utilising the Haploview software. The values in the boxes indicate the r^2^ values between the respective pairs of SNPs and the empty boxes represent those with r^2^ = 1.0. Haplotype blocks are defined by solid spine of linkage disequilibrium.
