# Supplementary material for: Genetic association between germline JAK2 polymorphisms and myeloproliferative neoplasms in Hong Kong Chinese population: a case–control study
Source: BMC Genet. 2014 Dec 20;15:147. doi: 10.1186/s12863-014-0147-y (PMC4293821; doi:10.1186/s12863-014-0147-y)
Supplement: Additional file 4: Table S2. — Summary of exhaustive haplotype analyses based on age- and sex-adjusted omnibus tests for sliding windows of up to 19 SNPs per window for 95 genotyped/imputed JAK2 SNPs for V617F-positive MPNs. [file 12863_2014_147_MOESM4_ESM.doc]

**Additional file 4:**

**Table S2.** Summary of exhaustive haplotype analyses based on age- and sex-adjusted omnibus tests for sliding windows of up to 19 SNPs per window for 95 genotyped/imputed *JAK2* SNPs for *V617F*-positive MPNs **^a^**

|  |  | SW with Omnibus Test *P_emp_* < 0.05 | | |  | Most Significant Omnibus Test | | |
| --- | --- | --- | --- | --- | --- | --- | --- | --- |
| No. | No. of SWs | No. of SWs | First SW ^b^ | Last SW ^b^ |  | SW | *P_asym_* | *P_emp_* |
| 1 | 95 | 74 | m5 | m95 |  | m43^*^ **^c^** | 3.76 × 10^-15^ | 2.00 × 10^-5^ |
| 2 | 94 | 88 | m3...m4^*^ | m94^*^...m95 |  | m42...m43^*^ | 8.76 × 10^-15^ | 2.00 × 10^-5^ |
| 3 | 93 | 92 | m1^*^...m3 | m93...m95 |  | m41...m43^*^ | 1.77 × 10^-14^ | 2.00 × 10^-5^ |
| 4 | 92 | 91 | m1^*^...m4^*^ | m92...m95 |  | m40...m43^*^ | 1.71 × 10^-14^ | 2.00 × 10^-5^ |
| 5 | 91 | 90 | m1^*^...m5 | m91...m95 |  | m42...m46 | 5.55 × 10^-14^ | 2.00 × 10^-5^ |
| 6 | 90 | 89 | m1^*^...m6 | m90...m95 |  | m38...m43^*^ | 7.19 × 10^-14^ | 2.00 × 10^-5^ |
| 7 | 89 | 88 | m1^*^...m7 | m89...m95 |  | m37...m43^*^ | 7.20 × 10^-14^ | 2.00 × 10^-5^ |
| 8 | 88 | 86 | m1^*^...m8 | m87...m94^*^ |  | m42...m49 | 2.09 × 10^-14^ | 2.00 × 10^-5^ |
| 9 | 87 | 86 | m1^*^...m9 | m87...m95 |  | m37...m45 | 5.84 × 10^-13^ | 2.00 × 10^-5^ |
| 10 | 86 | 85 | m1^*^...m10 | m86...m95 |  | m37...m46 | 5.84 × 10^-13^ | 2.00 × 10^-5^ |
| 11 | 85 | 84 | m1^*^...m11 | m85^*^...m95 |  | m37...m47^*^ | 1.65 × 10^-12^ | 2.00 × 10^-5^ |
| 12 | 84 | 82 | m1^*^...m12 | m84...m95 |  | m37...m48^*^ | 2.05 × 10^-12^ | 2.00 × 10^-5^ |
| 13 | 83 | 81 | m1^*^...m13 | m83...m95 |  | m37...m49 | 2.05 × 10^-12^ | 2.00 × 10^-5^ |
| 14 | 82 | 81 | m1^*^...m14^*^ | m82...m95 |  | m40...m53^*^ | 6.73 × 10^-12^ | 2.00 × 10^-5^ |
| 15 | 81 | 80 | m1^*^...m15 | m81...m95 |  | m38...m52 | 9.20 × 10^-12^ | 2.00 × 10^-5^ |
| 16 | 80 | 79 | m1^*^...m16 | m80...m95 |  | m38...m53^*^ | 7.44 × 10^-12^ | 2.00 × 10^-5^ |
| 17 | 79 | 78 | m1^*^...m17 | m79...m95 |  | m37...m53^*^ | 1.01 × 10^-11^ | 2.00 × 10^-5^ |
| 18 | 78 | 77 | m1^*^...m18 | m78...m95 |  | m38...m55 | 3.21 × 10^-11^ | 2.00 × 10^-5^ |
| 19 | 77 | 76 | m1^*^...m19 | m77...m95 |  | m39...m57 | 1.37 × 10^-11^ | 2.00 × 10^-5^ |

Abbreviations: SNP, single nucleotide polymorphism; SW, sliding window; *P_asym_*, asymptotic *P* value; *P_emp_*, empirical *P* value.

^a^  Note that this SNP identity is not the same as in **Table 1**. Please refer to **Table S2a** (see below) for the identity of the SNPs concerned.

The 95 genotyped/imputed *JAK2* SNPs were only tested for up to 19-SNP per SW as we fixed the comparison similar with that from the 19 genotyped SNPs for *V617F*-positive MPNs. The SW is shown as mx…my, where mx is the first SNP and my is the last SNP of the SW for the *JAK2* gene. Multiple comparisons were corrected by running 50,000 permutations to give the *P_emp_* value. The smallest *P_emp_* value generated after permutation is the same for all fixed-size SWs (2 × 10^-5^). The most significant results for each fixed-size SW is shown in the three rightmost columns. Note that, among all the 1634 SWs tested, m43 (see footnote c) always appears in the most significant SW.

^b^ Within each set of SWs with a given size (SNPs per SW), there are 1-2 SNPs or SWs that are *not* significant (*P_emp_*>0.05) and interrupt the consecutive significant SWs. Of particular note, there are 18 non-significant interrupting SWs for 1-SNP SW set, and 4 non-significant interrupting SWs for 2-SNP SW set.

^c^  m43^*^ is the same rs12342421 (S8). Of the 1634 SWs tested, m43^*^ alone gives the most significant result for association with *V617F*-positive MPNs among 95 *JAK2* SNPs.

**Table 2a.** SNP Identity of the 95 genotyped/ imputed *JAK2* SNPs

| No. | SNP | bp |  | No. | SNP | bp |  | No. | SNP | bp |
| --- | --- | --- | --- | --- | --- | --- | --- | --- | --- | --- |
| m1^*^ | rs3808850 (S1) | 4973311 |  | m33^*^ | rs1536798 (S5) | 5046931 |  | m65 | rs2274649 | 5080934 |
| m2 | rs1887429 | 4974549 |  | m34^*^ | rs10815148 (S6) | 5047284 |  | m66^*^ | rs3824432 (S16) | 5081675 |
| m3 | rs2274471 | 4975879 |  | m35 | rs11998913 | 5048048 |  | m67 | rs12340866 | 5084185 |
| m4^*^ | rs7849191 (S2) | 4978761 |  | m36^*^ | rs2149556 (S7) | 5049440 |  | m68 | rs3780370 | 5085167 |
| m5 | rs2225125 | 4988639 |  | m37 | rs1571437 | 5050334 |  | m69 | rs10974960 | 5085842 |
| m6 | rs1327494 | 4989303 |  | m38 | rs7859390 | 5052473 |  | m70 | rs7847141 | 5087171 |
| m7 | rs12347727 | 4990811 |  | m39 | rs913594 | 5053199 |  | m71^*^ | rs7847294 (S17) | 5087281 |
| m8 | rs4372063 | 4993338 |  | m40 | rs12339666 | 5053296 |  | m72 | rs3780372 | 5087544 |
| m9 | rs10115312 | 4993973 |  | m41 | rs10815149 | 5053701 |  | m73 | rs3780373 | 5088223 |
| m10 | rs7030260 | 4998070 |  | m42 | rs16922576 | 5054193 |  | m74 | rs10121077 | 5088411 |
| m11 | rs10815144 | 5000192 |  | m43^*^ | rs12342421 (S8) | 5055750 |  | m75 | rs3780374 | 5089677 |
| m12 | rs16922518 | 5002696 |  | m44 | rs3780365 | 5058520 |  | m76 | rs7870694 | 5090628 |
| m13 | rs10974914 | 5004332 |  | m45 | rs3780366 | 5058596 |  | m77 | rs10974963 | 5091305 |
| m14^*^ | rs7046736 (S3) | 5005732 |  | m46 | rs3780367 | 5058755 |  | m78 | rs2104685 | 5096023 |
| m15 | rs10974916 | 5007350 |  | m47^*^ | rs10974944 (S9) | 5060831 |  | m79 | rs4593605 | 5097278 |
| m16 | rs7034753 | 5011514 |  | m48^*^ | rs10119004 (S10) | 5061049 |  | m80 | rs10815157 | 5098771 |
| m17 | rs7851556 | 5012807 |  | m49 | rs4495487 | 5062798 |  | m81 | rs3780375 | 5099431 |
| m18 | rs11794708 | 5013441 |  | m50^*^ | rs10974947 (S11) | 5062846 |  | m82 | rs11793659 | 5099707 |
| m19 | rs7043489 | 5013604 |  | m51^*^ | rs12343867 (S12) | 5064189 |  | m83 | rs17425637 | 5100000 |
| m20 | rs11794778 | 5013794 |  | m52 | rs12349785 | 5066613 |  | m84 | rs3780377 | 5100899 |
| m21 | rs10974921 | 5014427 |  | m53^*^ | rs12340895 (S13) | 5066691 |  | m85^*^ | rs3780378 (S18) | 5102288 |
| m22 | rs2183137 | 5016293 |  | m54 | rs10815152 | 5066946 |  | m86 | rs3780379 | 5102519 |
| m23 | rs10974922 | 5018813 |  | m55 | rs12005893 | 5068046 |  | m87 | rs3824433 | 5103577 |
| m24 | rs7023146 | 5030163 |  | m56 | rs1159782 | 5068117 |  | m88 | rs884132 | 5104522 |
| m25 | rs7043371 | 5030203 |  | m57 | rs7875908 | 5071334 |  | m89 | rs3780381 | 5104523 |
| m26 | rs7037207 | 5033156 |  | m58 | rs7034539 | 5071585 |  | m90 | rs17425819 | 5104773 |
| m27 | rs7045491 | 5035658 |  | m59 | rs1410779 | 5073173 |  | m91 | rs10815160 | 5106616 |
| m28 | rs1328917 | 5039065 |  | m60^*^ | rs12343065 (S14) | 5073533 |  | m92 | rs11788963 | 5110157 |
| m29^*^ | rs2149555 (S4) | 5043743 |  | m61 | rs12348771 | 5073634 |  | m93 | rs966871 | 5111070 |
| m30 | rs1536800 | 5045434 |  | m62^*^ | rs7857730 (S15) | 5074049 |  | m94^*^ | rs10815162 (S19) | 5112291 |
| m31 | rs10974938 | 5046037 |  | m63 | rs6476939 | 5074837 |  | m95 | rs10974969 | 5115336 |
| m32 | rs10974939 | 5046482 |  | m64 | rs2031904 | 5077087 |  |  |  |  |

bp = base position
