# Supplementary material for: Genetic association between germline JAK2 polymorphisms and myeloproliferative neoplasms in Hong Kong Chinese population: a case–control study
Source: BMC Genet. 2014 Dec 20;15:147. doi: 10.1186/s12863-014-0147-y (PMC4293821; doi:10.1186/s12863-014-0147-y)
Supplement: Additional file 5: — Genotyping of JAK2 mutation and single nucleotide polymorphisms (SNPs). [file 12863_2014_147_MOESM5_ESM.doc]

**Additional file 5: Appendix S1**

**Genotyping of *JAK2* mutation and single nucleotide polymorphisms (SNPs)**

*Detection of JAK2 V617F mutation*

The method was modified from Jones *et al* . Amplification was performed in 96-well plates with a GeneAmp 9700 PCR System (Applied Biosystems) containing a 15-μL reaction volume with 0.3 unit HotStarTaq Plus DNA Polymerase (Qiagen), 1× PCR buffer (which contains KCl and (NH_4_)_2_SO_4_), 3.5 mM MgCl_2_, 0. 12 mM of each dNTP (deoxyribonucleoside triphosphate), 0.3 μM each of the forward and reverse outer primer, 0.5 μM of the forward wild type primer and 0.6 μM of the reverse mutant primers together with 30 ng of DNA. PCR conditions were 95°C for 5 minutes; 40 cycles with denaturation at 95°C for 30 seconds, annealing at 58°C for 45 seconds, and elongation at 72°C for 35 seconds; 1 cycle at 72°C for 5 minutes; and a final hold at 15°C. PCR products were pre-stained with SYBR Green I and then analysed by electrophoresis on 5% polyacrylamide gels at 130 V for 90 minutes. Sequencing was performed to confirm the mutation status in representative samples (BigDye Terminator v3.1; Applied Biosystems).

*Restriction fragment length polymorphism (RFLP) analysis*

Fourteen SNPs were genotyped by RFLP analysis (Additional file 6: **Table S3**). Polymerase chain reaction (PCR) was performed in a 10-μL reaction mixture containing 10 ng of genomic DNA, 2.5 mM MgCl_2_, 0.3 μM each of the forward and reverse primers, 0.2 mM each dNTP and 1× PCR buffer (which contains KCl and (NH_4_)_2_SO_4_) , 1.5 mM MgCl_2_ and 0.3 unit of HotStarTaq Plus DNA polymerase (Qiagen). Amplification was performed in 96-well plates with a GeneAmp 9700 PCR System (Applied Biosystems), including 1 cycle of initial denaturation for 5 minutes at 95°C, 30-35 cycles of 30 seconds at 95°C, 30 seconds at the annealing temperature (Additional file 5: Table S3), and 30 seconds at 72°C, plus 1 cycle of final extension for 5 minutes at 72°C. Digested products were pre-stained with SYBR Green I and then separated by electrophoresis in polyacrylamide gels of appropriate concentration .

*Unlabeled probe melting analysis*

The remaining five SNPs were genotyped by unlabelled probe melting analysis using the saturating dye SYTO 9 green fluorescent nucleic acid stain (final concentration of 2 μM; Invitrogen, Rockville, MD) . This method uses asymmetric PCR to generate single-stranded DNA (ssDNA) product. To the 3’ end of the unlabelled probe is added a phosphate group or a poly(A)/(T) tail to prevent probe extension. Asymmetric PCR was performed in a 10-μL reaction mixture containing 10 ng of genomic DNA, 2.5 or 3.5 mM MgCl_2_, 0.2 μM excess primer with a limiting:excess primer ratio of 1:10 (Additional file 5: Table S3), 0.2 mM each dNTP, 1× PCR buffer and 0.3 U of HotStarTaq Plus DNA Polymerase (Qiagen). Amplification was performed in 96-well plates with a GeneAmp 9700 PCR system, including 1 cycle of initial denaturation for 5 minutes at 95°C, 55 cycles of 30 seconds at 95°C, 20 seconds at the annealing temperature (Additional file 5: Table S3), and 20 seconds at 72°C, plus 1 cycle of final extension for 5 minutes at 72°C. After PCR, the probe and a saturating dsDNA dye were added to the ssDNA target for high-resolution melting analysis. A final 10-μL reaction mixture containing 9.1 μl of PCR product, 0.6 μM 3’-blocked probe (synthesised by IDT) and 2 μM SYTO 9 green fluorescent nucleic acid stain (Invitrogen) was prepared in 96-well white plates, and subjected to melting in LightCycler 480 Real-time PCR System (Roche). Probe/ssDNA amplicon duplexes were generated by heating samples to 95°C for 30 seconds, then cooling to 50°C for 30 seconds. The melting data were collected between 50°C and 95°C with a slope of 0.11°C/s at 5 acquisitions per °C, using the “melting-curves” analysis mode. Samples were again cooled to 40°C for 10 seconds and the melting curves were analysed with LightCycler 480 Software (Version 1.5, Roche).

**References**

1. Jones AV, Kreil S, Zoi K, Waghorn K, Curtis C, Zhang L, Score J, Seear R, Chase AJ, Grand FH, White H, Zoi C, Loukopoulos D, Terpos E, Vervessou EC, Schultheis B, Emig M, Ernst T, Lengfelder E, Hehlmann R, Hochhaus A, Oscier D, Silver RT, Reiter A, Cross NC: **Widespread occurrence of the JAK2 V617F mutation in chronic myeloproliferative disorders**. *Blood* 2005, **106**(6):2162-2168.

2. Jiang B, Yap MK, Leung KH, Ng PW, Fung WY, Lam WW, Gu YS, Yip SP: **PAX6 haplotypes are associated with high myopia in Han Chinese.** *PLoS One* 2011, **6**(5):e19587.

3. Mak JY, Yap MK, Fung WY, Ng PW, Yip SP: **Association of IGF1 gene haplotypes with high myopia in Chinese adults.** *Arch Ophthalmol* 2012, **130**(2):209-216.

4. Zhu MM, Yap MK, Ho DW, Fung WY, Ng P, Gu YS, Yip SP: **Investigating the relationship between UMODL1 gene polymorphisms and high myopia: a case-control study in Chinese.** *BMC Med Genet* 2012, **13**:64.

5. Yiu WC, Yap MK, Fung WY, Ng PW, Yip SP: **Genetic susceptibility to refractive error: association of vasoactive intestinal peptide receptor 2 (VIPR2) with high myopia in Chinese**. *PLoS One* 2013, **8**(4):e61805.

6. Zhou L, Myers AN, Vandersteen JG, Wang L, Wittwer CT: **Closed-tube genotyping with unlabeled oligonucleotide probes and a saturating DNA dye**. *Clin Chem* 2004, **50**(8):1328-1335.
