# Supplementary material for: Genetic association between germline JAK2 polymorphisms and myeloproliferative neoplasms in Hong Kong Chinese population: a case–control study
Source: BMC Genet. 2014 Dec 20;15:147. doi: 10.1186/s12863-014-0147-y (PMC4293821; doi:10.1186/s12863-014-0147-y)
Supplement: Additional file 6: Table S3. — JAK2 SNPs: Genotyping methods, primers, probes and key reaction conditions for PCR. [file 12863_2014_147_MOESM6_ESM.doc]

**Additional file 6:**

**Table S3.** *JAK2* SNPs: Genotyping methods, primers, probes and key reaction conditions for PCR

| SNP ^a^ | Genotyping method | Primer sequences (5’>3’) ^b^ | PCR conditions | | | | |
| --- | --- | --- | --- | --- | --- | --- | --- |
|  |  |  | Mg^2+^ (mM) | Primer (mM) | T_m_ (°C) | Cycles | Size (bp) ^c^ |
| rs3808850  (S1) | RFLP  (XmnI) | F: (T)_30_ GAA TTC CTT CTT TCC TGC AAA CAA AAA CTG  R: (T)_32_ CAC ATT TCC ATT TCC ACA GTT GTG AAG C | 2.5 | 0.3 | 62 | 35 | UC : 201  A : **75,** 18  T : **45, 30,** 18 |
| rs7849191  (S2) | UP | F: GTT GTA TTA GTC ACT TCC TGG  R: AAA TTA AGG GGT AGA AAA TGG TAA C  rP: TTA AGG AGA TGA ACA GAA GTA GAA GCA-phos | 2.5 | 0.2  0.02 | 55 | 55 | 115 |
| rs7046736  (S3) | UP | F: AGC CAA TTC GAG TCA CTT ATT C  R: CAG AAT TCC ATA GGC ACT CAG  rP: TTA GAA AAT GCG TTG ATG CTT TGC TAC GAA AAA- phos | 3.5 | 0.2  0.013 | 55 | 55 | 119 |
| rs2149555  (S4) | RFLP  (BseGI) | F: ACA TGC TTT CAA AGA GAG GCC AG  R: (T)_20_ ATT TTC CAT GCC GTA ATG TAT GCT AAT C | 2.5 | 0.3 | 62 | 35 | UC : 273  C : **216,** 57,  T : **133, 83,** 57 |
| rs1536798  (S5) | RFLP  (XceI) | F: (T)_9_ ATG GGA CTG TAT TTG GAC TTG GCT  R: (T)_27_ TGC ACA ATT CCA ACA ACA TGT CAG TAT AA | 2.5 | 0.3 | 60 | 35 | UC : 251  A : **208,** 43  C : **124, 84,** 43 |
| rs10815148  (S6) | RFLP  (SspI) | F: (T)_16_ GAT ACA TCA TGT TTC TTG CGG AAT ATT ATT CAT G  R: TGA CAG TGA AAA CAA AAG GTT ACT AAC ACA TAA TAC | 2.5 | 0.3 | 60 | 35 | UC : 204  T : **164,** 40  A : **101, 63,** 40 |
| rs2149556  (S7) | RFLP  (BseLI) | F: (T)_20_ GTC TAA ATG GAA TGA TAC TGT ATG TAC TCT TTT A  R: GTA AAT GTA TCC CTA TAA TCC CCA AAG TG | 2.5 | 0.3 | 60 | 35 | UC : 237  T : **192,** 45  C : **116, 76,** 45 |
| rs12342421  (S8) | UP | F: TAG ATC CTT ACT TCA ATA CTG GGT  R: ATA TTA AGC ATA GAC TAA ATC AAC TCT TT  fP: TCT CTT GGC TAG GAT GTG GTT TAT GTT GAC- phos | 2.5 | 0.02  0.2 | 55 | 55 | 240 |
| rs10974944  (S9) | RFLP  (BclI) | F: CTG TTC AAG GGT CAA CTG TAG TAC ATA AGA  R: (T)_30_ CTG GTT TTG ATC AGG GAT AGT CTC ACT T | 2.5 | 0.3 | 63 | 30 | UC : 208  G : **166,** 42  C : **101, 65,** 42 |
| rs10119004  (S10) | RFLP  (HphI) | F: (A)_12_ AAG TAA ATA CAT CCT CAG TAA AAC AAC ATA TAG AA  R: CAA GCA CCA CAC AAT ATT AGG AGA GTA | 2.5 | 0.3 | 60 | 35 | UC : 244  A : **194,** 50  G : **118, 76,** 50 |
| rs10974947  (S11) | UP | F: GAT TAA AAA AAT CAA TTC CAA ACT A  R: AAA TGT AAT TGT AGA GGA GC  fP: GTT TAG CAT TAT GTT AGG AGT GTT ATT ACT AAA AAA AT ^d^ | 3.5 | 0.02  0.2 | 50 | 55 | 207 |
| rs12343867  (S12) | RFLP  (TaiI) | F: (T)_26_ GGT TGA ACA TAA CGT TGG AAT AAC TG  R: ATA CTT TTA GTA GTC TCT GTG AAC ACC T | 2.5 | 0.3 | 60 | 35 | UC : 207  T : **169,** 38  C : **103, 66,** 38 |
| rs12340895  (S13) | RFLP  (BslI) | F: (T)_3_ TCA TGA CTT GCC TTA TTA TGG TAG TCT  R: (T)_10_ TAT ATA AAC AAT TTT CTT GAA TGT AAC CTT TGT G | 2.5 | 0.3 | 60 | 35 | UC : 122  G : **102,** 20  C : **62, 40,** 20 |
| rs12343065  (S14) | RFLP  (TasI) | F: (T)_10_ ACG ATA GTG GTA AGC TCT TTC TCG  R: (T) AAA AAG GGC AAG CAA ACA AAA ACC AAG | 2.5 | 0.3 | 60 | 35 | UC : 253  C : **145,** 60  T : **108, 85,** 60 |
| rs7857730  (S15) | UP | F: TTT TGA GAC ATA ATT TTA AGT GAA TAT AC  R: ATT TTA AGC TCA CGG AAC TAT G  fP: GTA ATT TTG AGT TAA TGT TCT TTT GCT TTT T- phos | 3.5 | 0.013  0.2 | 56 | 55 | 130 |
| rs3824432  (S16) | RFLP  (PagI) | F: GGT TCA TCA TTG TTA GTA TGT TTG TCG CAA  R: (T)_20_ GAC TTA AGC CTA TTC ATG AGC CAA AAT CTA | 2.5 | 0.3 | 60 | 32 | UC : 230  G : **192,** 38  A : **121, 71,** 38 |
| rs7847294  (S17) | RFLP  (SspI) | F: (T)_29_ TCC CAA TAT TAT ACA CCC CTT TTC GTC CAA C  R: GAG AGC ATC CCA AAG CCT GAT AGA ATA AG | 2.5 | 0.3 | 66 | 35 | UC : 260  C : **224,** 36  A : **136, 88,** 36 |
| rs3780378  (S18) | RFLP  (BseGI) | F: (T)_20_ GCC TGG GAG GAA CGT CGC A  R: CAG GAG TCG GAA CGC CAA GAG | 2.5 | 0.3 | 60 | 35 | UC : 386  T : **260,** 126  C : **181, 79,** 126 |
| rs10815162  (S19) | RFLP  (Bst1107I) | F: (T)_28_ TCC ACT AAG CCA AAA CGT TCC CTT GTA TA  R: (T)_8_ TTA AAG CTA TAA AAG AAA GAA CCT GTA TAC CAG AAG | 2.5 | 0.3 | 60 | 35 | UC : 172  G : **137,** 35  C : **82, 55,** 35 |

Abbreviations: SNP, single nucleotide polymorphism; RFLP, restriction fragment length polymorphism; UC, uncut; UP, unlabelled probe (melting analysis); and T_m_, annealing temperature for PCR.

^a^ The SNPs are shown in sequential order from the 5’ end to the 3’ end of the *JAK2* gene (sense strand).

^b^ F indicates forward primer, R reverse primer, and fP / rP forward / reverse unlabelled probes. A few primers have a poly-T tail at the 5’ end to enhance the size difference of restricted fragments for easy calling of genotypes. Note that all probes are phosphorylated (phos) at the 3’ end to prevent extension by DNA polymerase with one exception (Probe for rs10974947; see below). Underlined bases indicated mismatches to genomic templates and are created to prevent the formation of primer-dimers and/or hairpins.

^c^ For SNPs genotyped by RFLP, the allele that is recongisable by a specific restriction enzyme (RE) generates two distinct fragments (in **boldface**) which are produced by cleavage of a longer fragment (also in **boldface**) for the other allele that is *not* recognisable bythe same RE. The fragment present in both alleles is produced by cleavage of an internal restriction site. Please refer to **Additional file 7**: **Figure S7** for an example of RFLP patterns and **Additional file 8**: **Figure S8** for an example of melting curves obtained by unlabeled probe melting analysis.

^d^ A poly(A)/(T) tail is added to the 3’ end of the probe of rs10974947 (S11) to prevent extension by DNA polymerase. In fact, the addition of poly (A)/(T) tail was later adapted in our lab.
