# Supplementary material for: Genetic association between germline JAK2 polymorphisms and myeloproliferative neoplasms in Hong Kong Chinese population: a case–control study
Source: BMC Genet. 2014 Dec 20;15:147. doi: 10.1186/s12863-014-0147-y (PMC4293821; doi:10.1186/s12863-014-0147-y)
Supplement: Additional file 7: Figure S7. — SNP genotyping by restriction fragment length polymorphism. For illustration, rs10119004 (S10) is used as an example. A PCR fragment of 244 bp (see Additional file; Table S3) is amplified to encompass the SNP site. Upper panel: Restriction patterns for the two alleles of rs10119004 (S10) upon restriction digestion by the restriction enzyme HphI. Lower panel: Electrophoresis banding patterns on 8% polyacrylamide gel and stained with SYBR Green I. The DNA ladder is the 1 kb Plus DNA Ladder (lane M) from Invitrogen Life Technologies. [file 12863_2014_147_MOESM7_ESM.doc]

**Additional file 7: Figure S7**


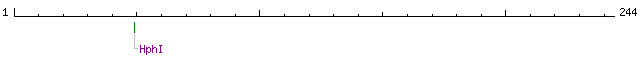

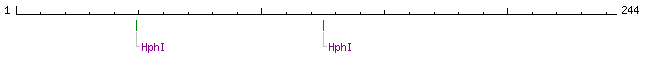


**A allele**

50 bp

194 bp

**G allele**

118 bp

76 bp

50 bp

194 bp

118 bp

76 bp

50 bp

**M**

**1 2 3 4 5 6 7 8**


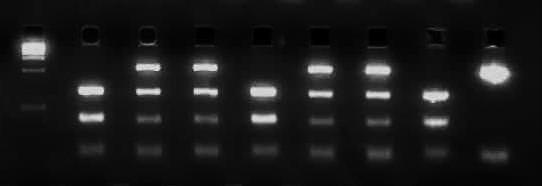


**1000 bp**

**100 bp**

**200 bp**

◄ Genotypes

**AG**

**AG**

**AG**

**GG**

**AA**

**AG**

**GG**

**GG**

1 Kb Plus DNA Ladder

**Additional file 7: Figure S7**. SNP genotyping by restriction fragment length polymorphism. For illustration, rs10119004 (S10) is used as an example. A PCR fragment of 244 bp is amplified to encompass the SNP site. **Upper panel:** Restriction patterns for the two alleles of rs10119004 (S10) upon restriction digestion by the restriction enzyme HphI. **Lower panel:** Electrophoresis banding patterns on 8% polyacrylamide gel and stained with SYBR Green I. The DNA ladder is the 1 kb Plus DNA Ladder (lane M) from Invitrogen Life Technologies.
