# Supplementary material for: Genetic association between germline JAK2 polymorphisms and myeloproliferative neoplasms in Hong Kong Chinese population: a case–control study
Source: BMC Genet. 2014 Dec 20;15:147. doi: 10.1186/s12863-014-0147-y (PMC4293821; doi:10.1186/s12863-014-0147-y)
Supplement: Additional file 8: Figure S8. — SNP genotyping by unlabelled probe melting analysis. For illustration, rs7849191 (S2) is used as an example. A PCR fragment of 115 bp (see Additional file 6: Table S3) is amplified to encompass the SNP site. The unlabelled probe is designed to match the C allele in this example. The probe-amplicon duplex of the homozygous genotype CC has a higher melting temperature than the probe-amplicon duplex of the homozygous genotype TT. Two peaks are obtained for the heterozygous genotype CT. [file 12863_2014_147_MOESM8_ESM.doc]

**Additional file 8: Figure S8**


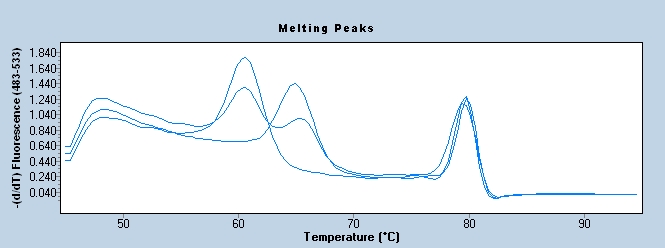


**CC**

Amplicon

**CT**

**TT**

**Additional file 8: Figure S8**. SNP genotyping by unlabelled probe melting analysis. For illustration, rs7849191 (S2) is used as an example. A PCR fragment of 115 bp (see **Additional file: Table S3**) is amplified to encompass the SNP site. The unlabelled probe is designed to match the C allele in this example. The probe-amplicon duplex of the homozygous genotype CC has a higher melting temperature than the probe-amplicon duplex of the homozygous genotype TT. Two peaks are obtained for the heterozygous genotype CT.
